# Inactivation of TRPM7 kinase in mice results in enlarged spleens, reduced T-cell proliferation and diminished store-operated calcium entry

**DOI:** 10.1038/s41598-018-21004-w

**Published:** 2018-02-14

**Authors:** Pavani Beesetty, Krystyna B. Wieczerzak, Jennifer N. Gibson, Taku Kaitsuka, Charles Tuan Luu, Masayuki Matsushita, J. Ashot Kozak

**Affiliations:** 1 Department of Neuroscience, Cell Biology and Physiology, Boonshoft School of Medicine, Wright State University, Dayton, OH 45435 USA; 2 Department of Molecular Physiology, Faculty of Life Sciences, Kumamoto University, Kumamoto, 860-8556 Japan; 3 Department of Molecular and Cellular Physiology, Graduate School of Medicine, University of the Ryukyus, Okinawa, 903-0215 Japan

## Abstract

T lymphocytes enlarge (blast) and proliferate in response to antigens in a multistep program that involves obligatory cytosolic calcium elevations. Store-operated calcium entry (SOCE) pathway is the primary source of Ca^2+^ in these cells. Here, we describe a novel modulator of blastogenesis, proliferation and SOCE: the TRPM7 channel kinase. TRPM7 kinase-dead (KD) K1646R knock-in mice exhibited splenomegaly and impaired blastogenic responses elicited by PMA/ionomycin or anti-CD3/CD28 antibodies. Splenic T-cell proliferation *in vitro* was weaker in the mutant compared to wildtype littermates. TRPM7 current magnitudes in WT and KD mouse T cells were, however, similar. We tested the dependence of T-cell proliferation on external Ca^2+^ and Mg^2+^ concentrations. At a fixed [Mg^2+^_o_] of ~0.4 mM, Ca^2+^_o_ stimulated proliferation with a steep concentration dependence and vice versa, at a fixed [Ca^2+^_o_] of ~0.4 mM, Mg^2+^_o_ positively regulated proliferation but with a shallower dependence. Proliferation was significantly lower in KD mouse than in wildtype at all Ca^2+^ and Mg^2+^ concentrations. Ca^2+^ elevations elicited by anti-CD3 antibody were diminished in KD mutant T cells and SOCE measured in activated KD splenocytes was reduced. These results demonstrate that a functional TRPM7 kinase supports robust SOCE, blastogenesis and proliferation, whereas its inactivation suppresses these cellular events.

## Introduction

Transient Receptor Potential Melastatin 7 (TRPM7) channel-kinase is highly expressed in cells of the immune system: lymphocytes, macrophages and mast cells^1–3^. TRPM7 protein is also expressed in many other cell types and tissues, albeit at lower levels. The channel activity of this protein is sensitive to cytoplasmic Mg^2+^, polyamines and pH^4^. In whole-cell patch clamp, TRPM7 current slowly develops as Mg^2+^ is depleted from cytosol^5^. Conversely, millimolar internal Mg^2+^ prevents current development. In inside-out patch configuration, single TRPM7 channels open sequentially when the cytosolic face of the membrane patch is rapidly exposed to Mg^2+^-free solutions, and can be recurrently inhibited by applying Mg^2+^. In Jurkat T lymphocytes, the inhibition of native TRPM7 channels by Mg^2+^ is biphasic with mean IC_50_-s of 10 µM and 165 µM^6^. Interestingly, with repeated exposure to Mg^2+^ the extent of inhibition of TRPM7 channels increases, indicating sensitization or use-dependence. Internal protons inhibit TRPM7 channels with IC_50_ of pH 6.3^4,6^. Inhibition by internal Mg^2+^, polyamines and protons is voltage-independent and in the case of Mg^2+^ reflects gradual reduction in the number of conducting (open) channels and a small step-like drop in unitary conductance^7^. Despite the high sensitivity of TRPM7 channels to Mg^2+^, significant basal currents are present in various cell types even before Mg^2+^ removal^8–10^. This observation is surprising, since the cytoplasmic [Mg^2+^] of ~1 mM^11^ would be sufficient to inhibit the majority of TRPM7 channels. Therefore, additional, positive regulators of this channel must be present in the cell. An obvious candidate is phosphatidyl inositol bisphosphate (PI(4,5)P_2_) phospholipid in the plasma membrane which stimulates TRPM7 as well as other TRP channels^4,12–14^.

TRPM7 channels are also sensitive to extracellular Mg^2+^ and Ca^2+^. Thus, in their presence the current-voltage (I-V) relation is steeply outwardly rectifying, whereas in their absence it is semi-linear^5,7,15^. The monovalent conductance of the TRPM7 channel differs in outward vs. inward direction, explaining the difference in current slopes seen in whole-cell recording. Extracellular divalent cations modify the I-V primarily by blocking the TRPM7 ion conduction pore which is permeable to Na^+^ and other monovalent cations^16,17^.

The kinase domain of TRPM7 belongs to the eukaryotic elongation factor 2 kinase (eEF-2K) family and functions as a serine/threonine kinase^18,19^. TRPM7 kinase is autophosphorylated, and was shown to phosphorylate phospholipase C (PLCγ2), annexin A1, myosins IIA- IIC and eEF-2K^20–26^. Recently, it was reported that under certain conditions this C-terminal kinase domain may be cleaved off and translocate to the nucleus, to participate in gene expression^27,28^. TRPM7 kinase activity is stimulated at high concentrations of Mg^2+^ but is not affected by Ca^2+^ ^4,23^. Moreover, the kinase domain has been suggested to play a role in cellular Mg^2+^ homeostasis: mice heterozygous for TRPM7 kinase deletion exhibited hypomagnesemia and reduced channel activity^29^.

Since the molecular identity of TRPM7 was discovered, two questions have been the focus of many studies: what is the relation of channel and kinase activities represented in the same polypeptide and what are the physiological roles of the channel vs. kinase in various cell types^13,18,30,31^. Cardiac-targeted TRPM7 deletion causes death due to congestive heart failure in mice^32,33^. Selective deletion of TRPM7 in metanephric mesenchyme in the mouse embryo causes defective nephrogenesis while selective deletion in neural crest causes disruption of pigment cell development, paralyzed hind legs and loss of large-diameter sensory neurons in the lumbar dorsal root ganglia^34^. Deletion of the entire gene or its channel and kinase portions individually, is embryonic lethal (embryonic age of 6–7 days)^2,29,34^. This has generally hampered the elucidation of TRPM7 function, at the same time emphasizing the importance of this protein for embryo development.

We recently characterized a new animal model of TRPM7: the kinase-dead mouse. It was constructed by introducing the K1646R point mutation into the kinase domain, rendering it inactive^10^. Importantly, such knock-in mice are born normally, have a normal lifespan and can be used to study the consequences of kinase inactivation on TRPM7 channel activity. We discovered that peritoneal macrophages express large TRPM7 currents in both wildtype (WT) and kinase-dead (KD) mutant animals. Interestingly, the basal TRPM7 channel activity was elevated in mutant mouse macrophages^10^. These initial experiments showed that the kinase function of TRPM7 is dispensable for its channel function and, in fact, may act to suppress channel activity in intact macrophages. The mechanism by which channel basal activity is stimulated in kinase-dead mouse macrophages is currently not known.

In the present study we investigated in detail the consequences of TRPM7 kinase inactivation in the mouse spleen. We found that KD mutant mice exhibit splenomegaly. Both Ca^2+^ and Mg^2+^ strongly influence T-cell proliferation and are thought to permeate TRPM7^35^. Blastogenesis (enlargement) and proliferation of splenic T lymphocytes in response to PMA/ionomycin and anti-CD3/CD28 stimulation *in vitro* were, therefore, measured in WT and KD mutant mice at various Ca^2+^_o_ and Mg^2+^_o_ concentrations. In the presence of fixed maximum concentration of one of the cations (approximately 400 µM), the other cation stimulated proliferation in a concentration-dependent manner. After 24 hrs of stimulation with phorbol myristate acetate (PMA) and ionomycin, the KD mutant T cells proliferated less than wildtype at all tested Ca^2+^_o_ and Mg^2+^_o_ concentrations (1–400 µM). At 48 hrs of stimulation, the differences between WT and KD were smaller at lower divalent cation concentrations whereas at high Ca^2+^_o_ or Mg^2+^_o_, proliferation rates were essentially the same. The Ca^2+^ and Mg^2+^ EC_50_ values for KD lymphocytes were also somewhat higher compared to WT. Measured in patch clamp, TRPM7 current amplitudes in KD mutant T cells were not different from WT. Reverse transcriptase polymerase chain reaction (RT-PCR) experiments performed with purified splenic T-cell RNA showed that in addition to TRPM7, these cells also express TRPM6 mRNA, a closely related channel-kinase, but very little TRPV1, a Ca^2+^-permeable channel that was recently reported in murine T cells. TRPM7 protein was detected in Western blots and its expression was increased in activated T cells of both WT and KD animals. Neither TRPM7 nor TRPM6 mRNA were up-regulated during activation, however. Immunosuppressive drugs cyclosporine A (CsA) and FK506 did not alter TRPM7 and TRPM6 mRNA expression, ruling out dependence on the nuclear factor of activated T cells (NFAT). T-cell diameters were measured in resting and 24–96 hr stimulated cells using an automated cell counter. The KD mutant T cells exhibited reduced blastogenesis compared to WT. Paradoxically, resting T-cell diameters were slightly larger in KD compared to WT mice. Since store-operated calcium entry through Orai/STIM (stromal interacting molecule) channels plays a crucial role in T-cell activation and proliferation^36,37^, we measured SOCE in single resting and PMA/ionomycin activated splenocytes. Both basal cellular calcium levels and SOCE were reduced in activated KD splenocytes compared to WT controls, with no change in the Ca^2+^ store content. By contrast, SOCE rate of rise in resting (unstimulated) KD cells was modestly increased. Acute stimulation of purified T cells by anti-CD3 crosslinking resulted in Ca^2+^ elevations that were smaller in KD mutant than in WT. We conclude from these experiments that in activated T lymphocytes, TRPM7 kinase deficiency leads to suppression of SOCE, blastogenesis and proliferation.

A preliminary report of this work has appeared^38^.

## Results

### TRPM7 KD mutant mice exhibit splenomegaly

**Figure 1:**
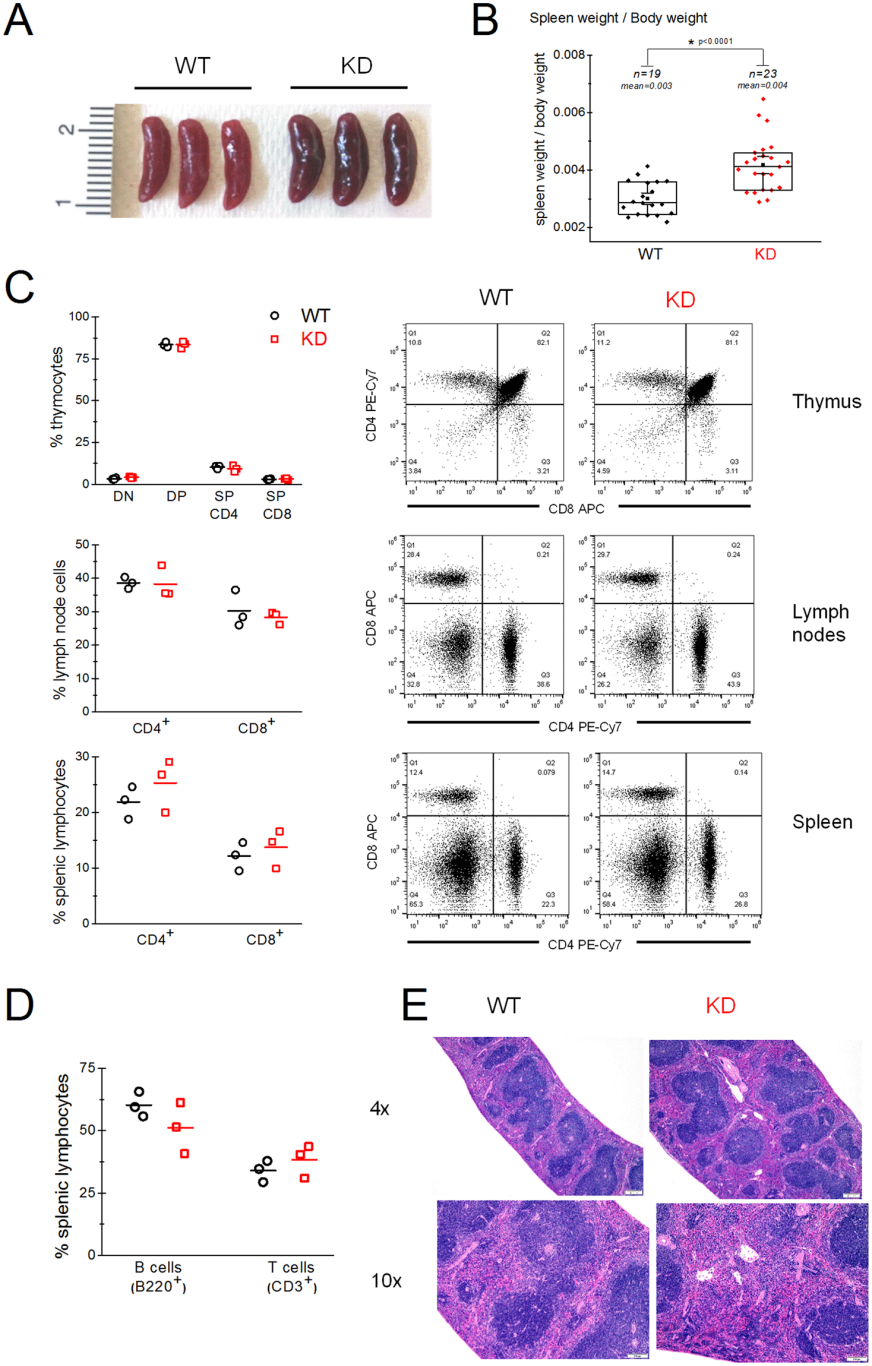
Spleens of TRPM7 kinase-dead animals are enlarged. (**A**) Photograph of spleens isolated from WT (left) and TRPM7 KD (right) mice. (**B**) Spleen weight/mouse weight ratios in WT and KD mutant. “n” indicates the number of mice in each group. (**C**) Flow cytometric analysis of thymocytes, lymph node cells and lymphocyte population of splenocytes for the relative percentage of CD4^+^ and CD8^+^ cells in WT and KD mice. DN-double negative (CD4^−^, CD8^−^), DP-double positive (CD4^+^, CD8^+^), SP-single positive (CD4^+^, CD8^−^ or CD4^−^, CD8^+^). (**D**) B and T cell profile of splenic lymphocyte population as determined by B220 FITC (B cells) and CD3 PE-Cy7 (T cells). (**E**) Hematoxylin-eosin (HE) staining of spleen sections from WT and KD mice at 4× and 10× magnification. (**C**,**D**) Data from two independent experiments. Horizontal lines represent arithmetic means. (**C**–**E**) Data collected from 3 WT and 3 KD mice. (**A**–**E**) Mouse ages were 2.5–3 months.

Our initial characterization of TRPM7 kinase-dead mice included body weight measurements which were in the normal range^10^. We found, however, that spleens isolated from KD mutant were significantly larger in size with a mean weight of 0.07 g (±0.002) for WT (19 mice) vs. 0.10 g (±0.005) for KD mutant (23 mice) (Fig. 1). We normalized spleen weights to animal weights in order to rule out the possibility that only heavier mice were selected for analysis. Even after normalization, the spleens of the KD mutant mice were still larger by 33.3% (Fig. 1B). The absolute numbers of splenocytes from KD mice were also increased by about 37%, a rough estimate based on 3 WT and 3 KD mice (data not shown). Flow cytometric investigation of thymus gland, lymph nodes and spleen showed no significant differences between WT and KD in percentage composition of CD4^+^ and CD8^+^ T cell subsets (Fig. 1C). The percentage of B cells and T cells in the KD splenic lymphocyte population were also normal (Fig. 1D). Histological examination of KD mouse spleens showed a mild, diffuse increase in extramedullary hematopoiesis (EMH) within the red pulp, with an increase in erythroid, myeloid and megakaryocytic precursors (Fig. 1E). However, complete blood counts with white blood cell differentials showed no overt abnormalities in erythrocytes, leukocytes and platelets compared to WT (Supplementary Table S1).

### Proliferation of splenic T lymphocytes is stimulated by external Ca^2+^ and Mg^2+^ and is impaired in TRPM7 KD mutant

**Figure 2:**
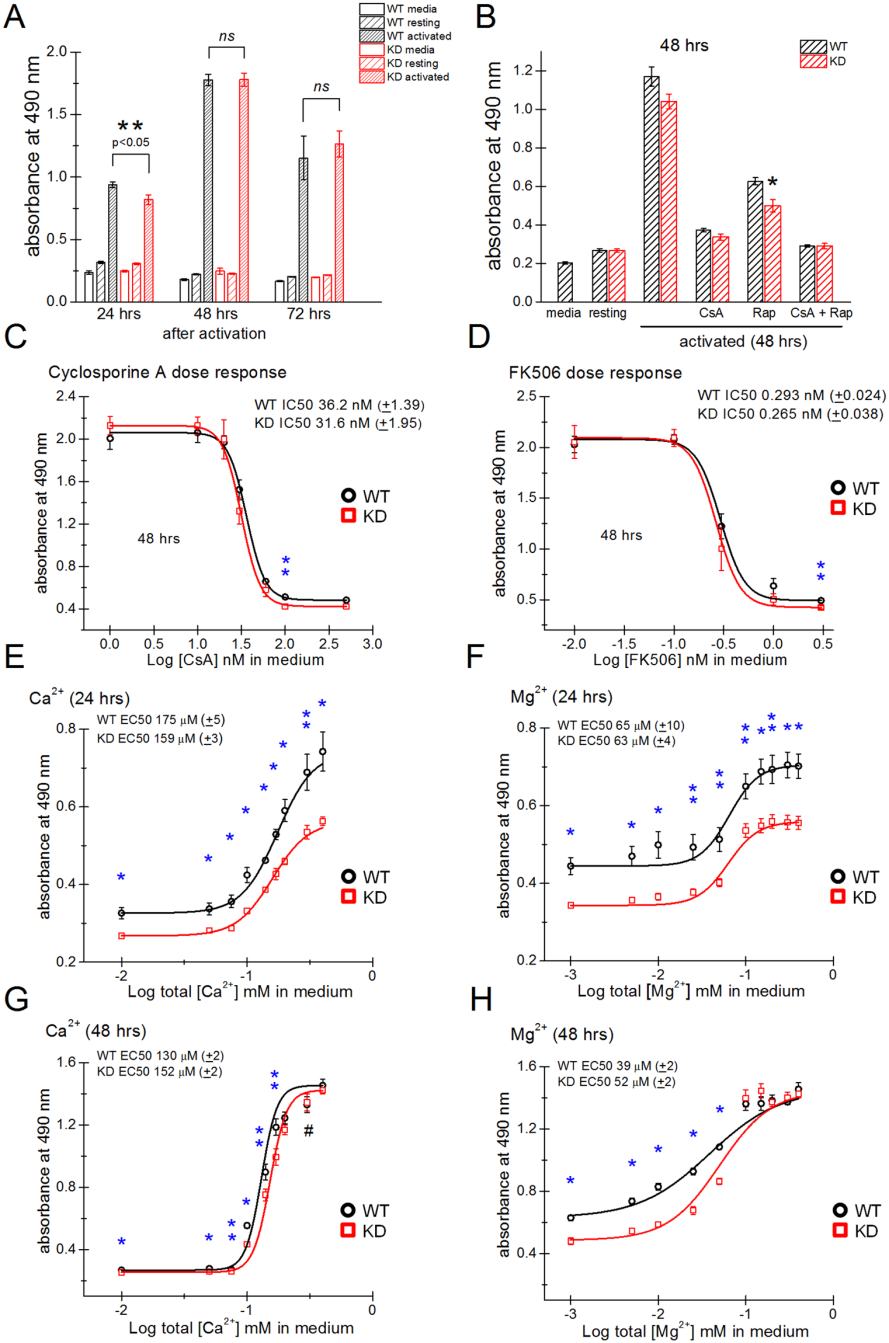
Dependence of WT and TRPM7 KD mouse splenic T-lymphocyte proliferation on extracellular [Ca^2+^] and [Mg^2+^]. (**A**) T-cell proliferation measured using MTS-based colorimetric assay after 24, 48 and 72 hrs of PMA/ionomycin stimulation. (**B**) The sensitivity of WT and KD T-cell proliferation to CsA (500 nM) and/or rapamycin (300 nM) was measured 48 hrs after activation. (**C**,**D**) Cyclosporine A and FK506 dose response relations for WT and KD T-cell proliferation. (**E**,**G**) Dependence of proliferation rate on external [Ca^2+^] at a fixed [Mg^2+^] of 0.406 mM measured at 24 and 48 hrs, respectively. (**F**,**H**) Dependence of proliferation rate on external [Mg^2+^] at a fixed [Ca^2+^] of 0.424 mM measured at 24 and 48 hrs, respectively. Note that there is significant proliferation even at 1 µM [Mg^2+^]. In (**E**–**H**) RPMI medium was treated with Chelex-100 resin prior to adding CaCl_2_ and MgCl_2_ at indicated concentrations. *p < 0.01, **p < 0.05. In (**C**–**H**) data were fitted with dose response function using OriginLab. In (**A**–**H**): activation was performed using PMA/ionomycin. Data collected from at least 3 WT and 3 KD mutant mice. For # in (**G**), data was collected from 2 KD mice.

As TRPM7 is one of the six main channel types expressed in human T cells^39^, we hypothesized that the function of splenic T lymphocytes may be altered in the KD mutant. As an initial characterization, we measured the proliferation rates of purified T cells from both WT and KD mutant mice using the MTS colorimetric assay, in which absorbance measurements (at 490 nm) correspond to the number of metabolically active cells (see *Materials and Methods*). T cells were stimulated with PMA, a phorbol ester, and the calcium ionophore ionomycin^40^. The plate reader-based assay was performed at various time points after the addition of mitogen. These experiments revealed that KD mutant lymphocyte proliferation was reduced by 13% compared to WT at 24 hours. The mean absorbance values for WT were 0.94 ± 0.02 and for KD were 0.82 ± 0.04. At 48 and 72 hours, however, no significant difference was observed between WT and KD T-cell proliferation (Fig. 2A). Under similar activation conditions, the viable cell densities, counted using a hemocytometer were also lower at 24 hours for KD T cells compared to WT. However, at 48 hours cell densities were not significantly different (Supplementary Fig. S1).

Next, we compared the ability of immunosuppressants, CsA and rapamycin to inhibit proliferation in WT and KD T cells. CsA is a calcineurin inhibitor thought to act by suppressing the Ca^2+^-dependent NFAT transcription factor, whereas rapamycin is an inhibitor of mTOR (mechanistic target of rapamycin), a serine/threonine kinase^41,42^. We found that after 48 hours of stimulation the KD mutant T-cell proliferation was more sensitive to rapamycin inhibition than WT (Fig. 2B) but at 24 hrs the difference was not significant (Supplementary Fig. S1). We tested if the NFAT pathway was defective in the KD by plotting the dependence of WT and KD T-cell proliferation on calcineurin inhibitors CsA (tested at 1–500 nM) and FK506 (0.01–3 nM). As shown in Fig. 2C and D, KD T cells had a tendency to proliferate less compared to WT at higher concentrations of CsA and FK506 but the difference was significant only for 100 nM CsA and 3 nM FK506. The IC_50_ values for CsA (WT: 36 nM ± 1.4, KD: 32 nM ± 1.9) and FK506 (WT: 0.29 nM ± 0.02, KD: 0.26 nM ± 0.04) were not different between WT and KD (Supplementary Table S2). These results suggest that the NFAT-dependent pathways may be only slightly suppressed in KD T cells.

TRPM7 has been reported to conduct both Ca^2+^ and Mg^2+^ as well as other metal ions such as Zn^2+^ ^43^. Both Ca^2+^ and Mg^2+^ have significant and well-documented roles in T-lymphocyte proliferation (e.g.^44–48^). Ca^2+^ entry in lymphocytes occurs chiefly through Orai/STIM channels which are activated by endoplasmic reticulum Ca^2+^ store depletion downstream of T-cell receptor (TCR) engagement. Recently, it was reported that TRPV1 may also participate in murine T-cell Ca^2+^ signaling^49,50^. Mg^2+^ entry pathways of lymphocytes (and other cell types) are less well characterized but may involve the Mg^2+^ transporter MagT1^48,51,52^. In view of importance of these ions for T-cell function, we investigated the dependence of proliferation on extracellular Ca^2+^ and Mg^2+^ in C57BL/6 mice. Roswell Park Memorial Institute (RPMI)1640 medium, commonly used for culturing lymphocytes, contains 0.424 mM total Ca^2+^ and 0.406 mM total Mg^2+^. By treating with Chelex-100 resin, we modified the RPMI medium to keep either Ca^2+^ or Mg^2+^ at its maximal (i.e. 0.424 or 0.406 mM) concentration and systematically vary the concentration of the complementary divalent cation. Cell population proliferation readings were taken after 24 and 48 hrs of stimulation with PMA/ionomycin in these media. Proliferation was consistently higher when T cells were stimulated for 48 hrs compared to 24 hrs at PMA concentrations of 10–250 ng/ml, with ionomycin concentration kept at 250 nM (Supplementary Fig. S1). Proliferation rates at 24 hrs were significantly reduced in the KD mutant compared to WT for all Ca^2+^ concentrations tested (Fig. 2E). The same was true for Mg^2+^ (Fig. 2F). At 48 hrs, the rates were increased for both WT and KD but for KD remained significantly lower than WT at 10–170 µM [Ca^2+^] or 1–50 µM [Mg^2+^] (Fig. 2G,H). The difference between WT and KD was overcome by increasing extracellular [Ca^2+^] or [Mg^2+^] above 170 µM and 50 µM, respectively.

24 hours after activation, the EC_50_ values for Ca^2+^ were 175 µM and for Mg^2+^ 65 µM in WT T cells. The Mg^2+^ EC_50_ value for KD T cells was 63 µM, not different from WT, however the Ca^2+^ EC_50_ was slightly lower in the KD T cells (159 µM). 48 hours after activation, the EC_50_ values for Ca^2+^ and Mg^2+^ were reduced compared to 24 hrs in WT (Ca^2+^ 130 µM; Mg^2+^ 39 µM), but such a robust shift in EC_50_ values was not observed for KD T cells (Ca^2+^:152 µM, Mg^2+^:52 µM) (Fig. 2E–H and Supplementary Table S2).

As shown in Fig. 2E–H, the slopes of the fitted curves, determined by Hill coefficient, were steeper for Ca^2+^ in both WT (6.96 ± 0.32) and KD (6.50 ± 0.20) at 48 hours compared to 24 hours (WT: 3.04 ± 0.38, KD: 3.03 ± 0.19) without a major difference. The slope of Mg^2+^ dependence became shallower at 48 compared to 24 hours. At 24 hours, the slopes were similar for WT (2.62 ± 0.81) and KD (2.53 ± 0.38) but at 48 hours (WT: 1.08 ± 0.06, KD: 1.46 ± 0.06), the reduction in slope was smaller in KD (Fig. 2E–H and Supplementary Table S2). The Ca^2+^ dose response curves were steeper than Mg^2+^ at 48 hours after activation (Fig. 2G,H and Supplementary Table S2). These results suggest that the Ca^2+^ and Mg^2+^ influx pathways which are upregulated in WT T cells upon activation, are hampered in KD T cells. The defect could be in the upregulation or in the functional kinetics of these pathways. These experiments showed that genetic inactivation of TRPM7 kinase results in reduced proliferation of splenic T cells upon mitogenic stimulation, and that this defect was ameliorated at high Ca^2+^ or Mg^2+^ concentrations in the bathing medium.

### Blastogenesis is suppressed in TRPM7 KD mutant mouse T cells

**Figure 3:**
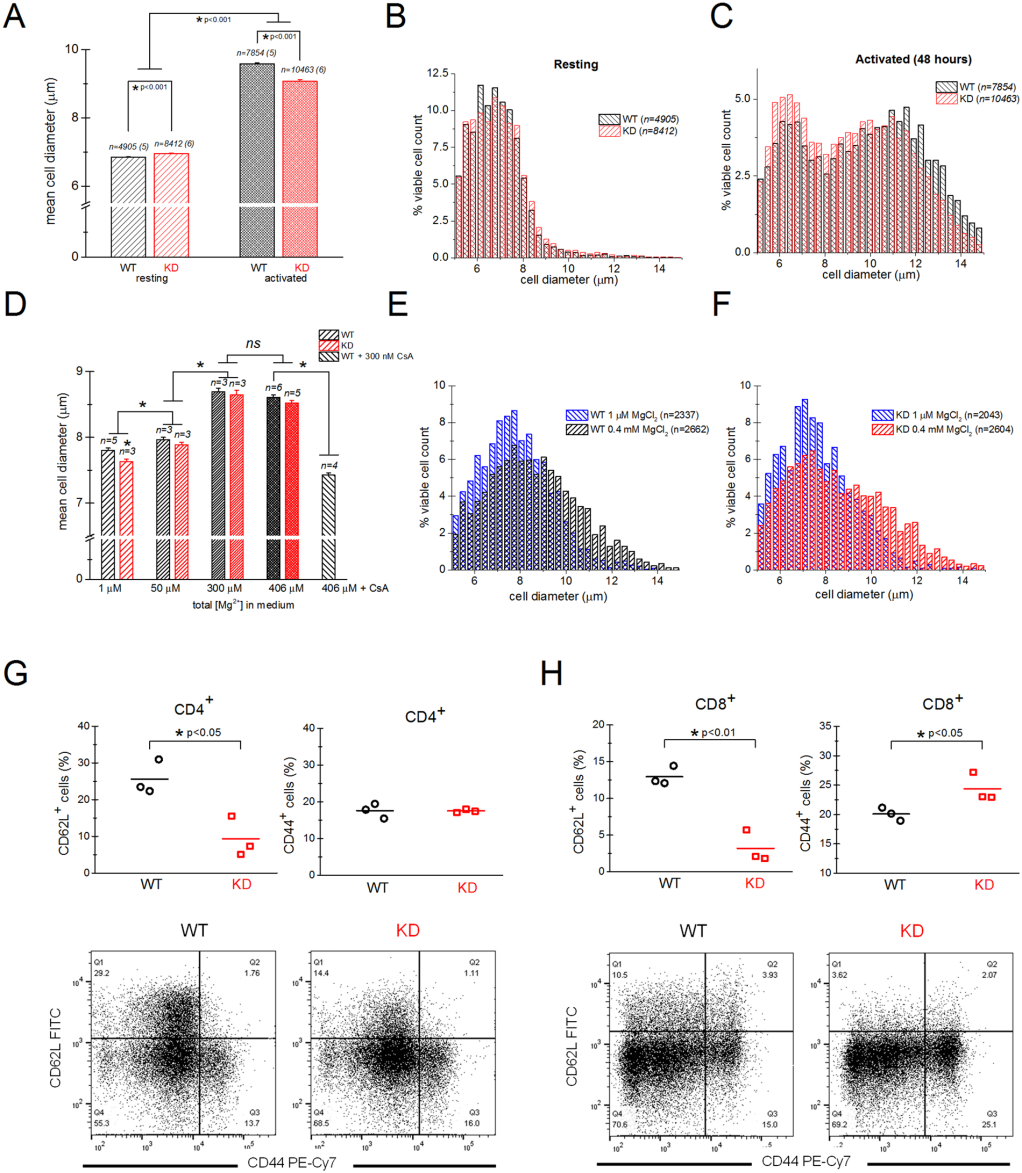
Dependence of T-cell blastogenesis on external [Mg^2+^]. (**A**) Mean cell diameters of WT and KD mouse T cells at rest and 48 hours after activation with PMA/ionomycin in normal RPMI containing 0.424 mM Ca^2+^ and 0.406 mM Mg^2+^. (**B**,**C**) Histograms of resting (**B**) and activated T-cell diameters (**C**). Same data as in bar plot in (**A**). (**D**) Diameters of T cells treated with PMA/ionomycin were measured after 24 hrs activation in chelex-treated RPMI medium supplemented with indicated MgCl_2_ concentrations. [Ca^2+^] was fixed at 0.424 mM. *p < 0.001. (**E**,**F**) Histograms of WT and KD T-cell diameters in Chelex-treated RPMI with 1 µM and 0.4 mM MgCl_2_ added to the medium. Same data as in bar plot in (**D**). In A the number of mice is given in parentheses. In (**D**) “n” indicate the number of mice used. For (**A**–**F**) (except **D**) “n” indicates the number of cells. (**G**,**H**). Analysis of CD62L and CD44 expression by CD4^+^ (**G**) and CD8^+^ (**H**) T cells from 11–12 wk old WT and KD spleens, determined by flow cytometry. Collective data from 3 mice are shown as scatter plots and representative flow cytometry dot plots are presented beneath. Horizontal lines represent arithmetic means.

Upon activation, T cells increase their volume by a process termed blastogenesis or blast transformation, which is followed by cell division. The blastogenic response directly reflects the extent of T-cell activation (see^53^ and references within). In order to examine in detail the defect in KD mouse T-cell proliferation, we compared the diameters of T cells isolated from WT and KD mutant mice under resting and PMA/ionomycin stimulated conditions. This is a single-cell measurement and can reliably detect blastogenesis in mitogen-stimulated cells as well as the effects of immunosuppressive drugs in preventing blastogenesis. Importantly, it reports actual cell sizes without an upper limit dictated by the reporter system itself, as is the case with colorimetric assays^53^. Upon activation with PMA/ionomycin for 48 hours, the blastogenic response was observed in both WT and KD T cells but it was smaller in KD (Fig. 3A–C). Activation resulted in a much smaller increase in diameter (30%) and volume (121%) in KD T cells than in WT T cells (40% increase in diameter and 173% increase in volume) (Fig. 3A–C). These results are in accordance with the reduced proliferation observed in normal RPMI (see Fig. 2A).

Next, we compared mean cell diameters under increasing Mg^2+^_o_ concentrations. Mg^2+^stimulated blastogenesis in a concentration-dependent manner (Fig. 3D–F), in agreement with our proliferation data (Fig. 2F,H). As expected, blastogenesis was substantially reduced when CsA was added to the medium, emphasizing the contribution of NFAT-dependent pathways to cell enlargement (Supplementary Fig. S2)^53^. Blastogenesis was slightly reduced in the KD mutant mouse and this effect became even more significant at low Mg^2+^ concentrations (1 µM) (Fig. 3D and Supplementary Fig. S2). Viability of WT and KD mouse T cells at various concentrations of Mg^2+^ was not significantly different (Supplementary Fig. S2) suggesting that the proliferation defects observed in KD T cells (Fig. 2F,H) at various [Mg^2+^]_o_ do not reflect increased KD T-cell death. Interestingly, the stimulatory effect of Mg^2+^ on blastogenesis essentially saturated at 300 µM, below 400 µM present in normal medium (Fig. 3D).

In young (13–18 weeks; Supplementary Fig. S3) and older animals (4–12 months; Fig. 3A), the resting T-cell mean diameter was larger in KD mutant compared to WT: by 0.76% in younger mice and 1.31% in older mice which corresponds to 2.5% and 4.3% increase in volume, respectively. Increases in resting T-cell diameters have been reported in interleukin-2 (IL2) deficient mice and LAT Y136F knock-in mice. These cells also expressed activation markers CD44 and/or CD69 and had reduced expression of IL-2Rα or CD45 RB suggesting a pre-activated state^54,55^. CD4^+^ T cells from KD mice showed diminished expression of CD62L (L-selectin) suggesting an activated state (Fig. 3G). Similar phenotype was also seen in KD CD8^+^ T cells along with an upregulation of CD44, a marker for previously activated memory T cells (Fig. 3H). Approximately 16% of CD8 T cells showed effector/memory phenotype (CD62L^−^CD44^+^) in WT, whereas this percentage increased to about 23% in KD mice. We also detected a ~9% lower expression of CD3 in KD T cells compared to WT, as measured by median fluorescence intensity, which was not, however, statistically significant (data not shown). Collectively, cell size measurement and surface marker expression data suggest that KD mouse T cells display a “hyperactivated” phenotype.

### Expression of TRPM7 and TRPM6 in T lymphocytes

**Figure 4:**
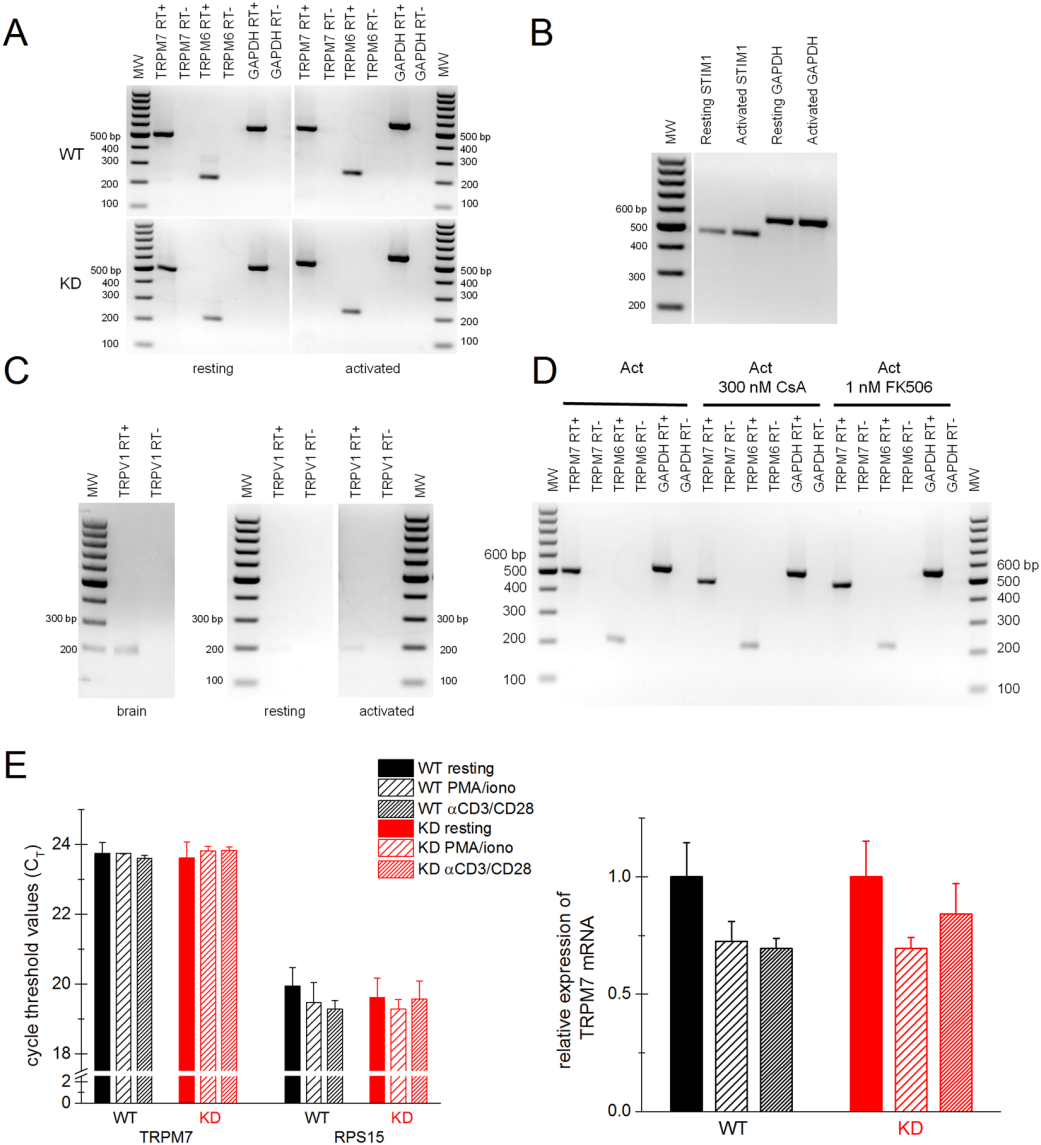
TRPM7 is highly expressed in WT and KD mutant resting and activated splenic T cells. (**A**) Expression of mTRPM7, mTRPM6 and glyceraldehyde 3-phosphate dehydrogenase (GAPDH) in resting and activated WT and KD T cells. PCR cycles: 38. (**B**) RT-PCR of STIM1 and GAPDH in WT T cells. PCR cycles: 27. The RT-PCR reactions without reverse transcriptase (RT-) did not amplify any DNA fragment as expected (data not shown). (**C**) RT-PCR of TRPV1 in WT T cells and in WT brain as a positive control (38 PCR cycles). For (**A**–**C**) the RNA used for RT-PCR was isolated from WT and KD mouse purified T cells that were quiescent or activated for 48 hrs with PMA/ionomycin. The white gap in the center of (**A**) and (**C**) indicates different gels or spliced lanes in the same gel. In (**B**), the white gap after the molecular weight (MW) marker lane indicates spliced and combined lanes of the same gel. (**D**) RT-PCR of mTRPM7, mTRPM6 and GAPDH was performed on total RNA isolated from WT T cells, 48 hrs after activation with PMA/ionomycin in the presence and absence of 300 nM CsA or 1 nM FK506. Number of PCR cycles: 35. The primer sequences are provided in Supplementary Table S2. (**E**). RT-qPCR analysis of TRPM7 gene expression in resting and activated WT and KD T cells. C_T_ values (error bars represent SD) are shown in the left panel; the relative expression of TRPM7 in activated T-cell samples, with resting TRPM7 expression levels normalized to 1, are shown in the right panel (error bars represent SEM). RPS15 was used as an internal control. Data were collected from three independent experiments with total 3 WT and 3 KD mice.

TRPM7 is the most abundant TRP channel expressed in human CD4+ T cells^56^. TRPM7 expression has also been reported in murine T lymphocytes^1,2,57^. In order to characterize TRPM7 expression in KD splenic T cells compared to WT, we performed reverse transcriptase PCR (RT-PCR) and Western blot experiments with purified T cells. TRPM7 was highly expressed in T cells at the mRNA level (Fig. 4A) in both WT and KD. Surprisingly, TRPM6, a closely related channel-kinase also showed a strong signal in RT-PCR. We investigated if TRPM7 or TRPM6 mRNA levels are changed by PMA/ionomycin treatment, as is the case for numerous T-cell specific genes. However, we found no major difference between resting and activated lymphocytes (Fig. 4A), consistent with the expression of TRPM7 observed in human CD4^+^ T cells upon stimulation with anti-CD3/anti-CD28 coated beads^56^. By contrast, STIM1 expression was increased upon activation in WT T cells, in agreement with previous reports in stimulated human T lymphocytes (Fig. 4B)^56,58^.

Recently, TRPV1, a thermosensitive channel belonging to the TRPV subfamily of TRP channels, was reported to contribute to calcium signaling resulting from T-cell receptor engagement^49,50^. We, therefore, also performed RT-PCR with TRPV1-specific primers (Supplementary Table S2). In WT resting and activated T cells there was only a faint band corresponding to TRPV1, suggesting that this channel is not a major contributor to Ca^2+^ signaling in murine splenic T cells (Fig. 4C). In control experiments this set of primers recognized murine brain TRPV1 (Fig. 4C). This is in agreement with^57^  that did not find TRPV1 mRNA expression in murine splenocytes.

Because NFAT is a major regulator of gene expression during T-cell activation process, we tested if it would also regulate the expression of TRPM7 and TRPM6. However, RT-PCR performed on T cells activated in the presence of calcineurin inhibitors CsA and FK506 did not reveal any major difference in their expression levels (Fig. 4D). This finding is in agreement with lack of effect of these drugs on TRPM7 and TRPM6 mRNA expression in Caco2 epithelial cell line^59^.

We further confirmed the RT-PCR results of TRPM7 expression in T cells using quantitative real-time PCR (RT-qPCR). As shown in Fig. 4E, TRPM7 mRNA expression in resting T cells was not different between WT and KD. The expression did not significantly change upon PMA/ionomycin or anti-CD3/CD28 stimulation in both WT and KD T cells, in agreement with our RT-PCR data.

**Figure 5:**
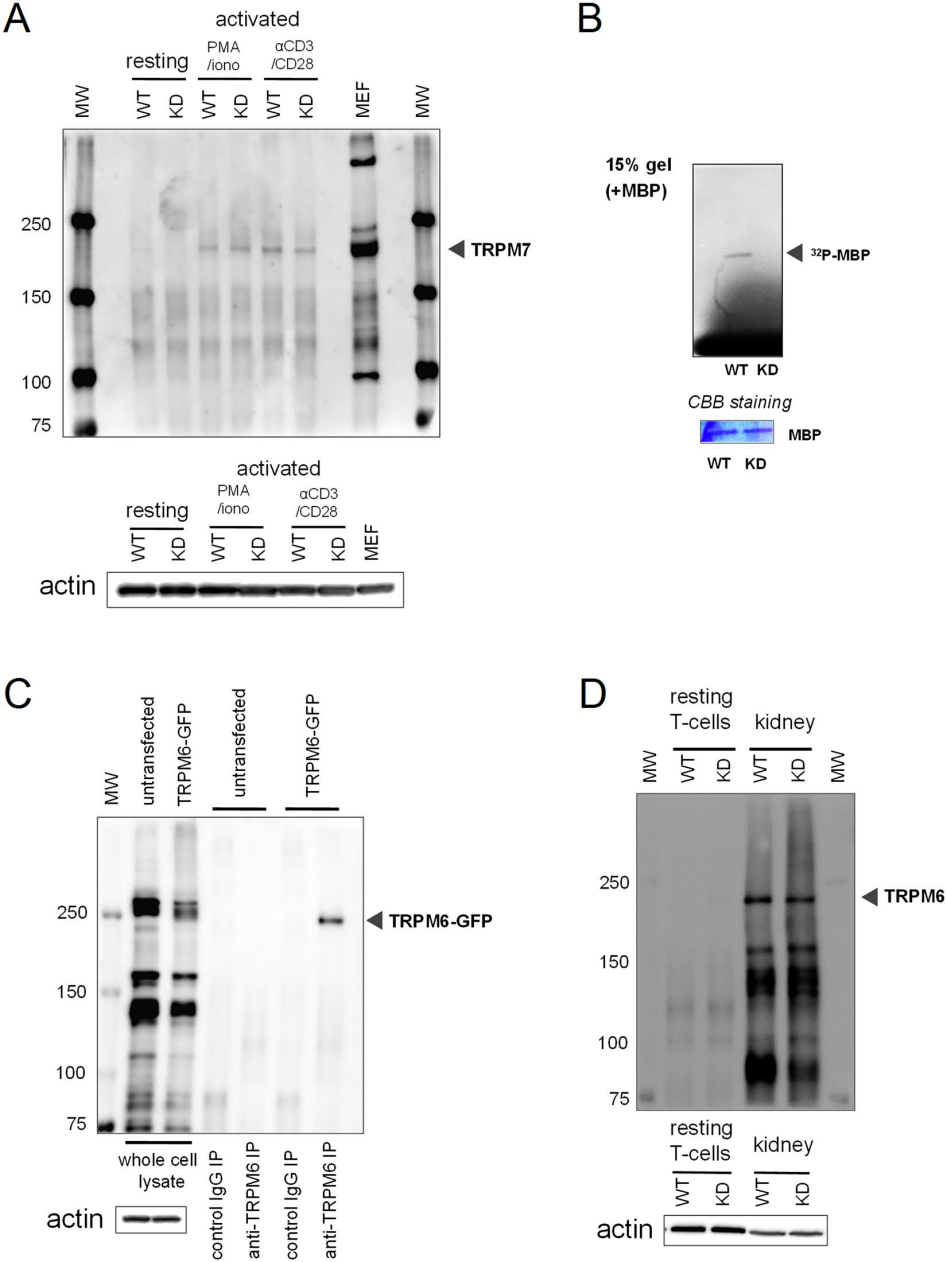
TRPM7/TRPM6 protein expression and TRPM7 kinase activity in splenic T cells. (**A**) Western blot analysis of immunoprecipitated TRPM7 from whole cell lysates of WT and KD splenic T cells. T cells were stimulated with PMA/ionomycin or anti-CD3/CD28 antibody coated beads for 48 hrs. Mouse embryonic fibroblasts were used as a positive control. Equal amounts of protein before immunoprecipitation were ensured by probing for actin. (**B**) Incorporation of ^32^P into exogenous myelin basic protein (MBP) by TRPM7 immunoprecipitated from WT and KD resting T cells. Equal quantities of MBP were verified by coomassie blue staining. (**C**) Control experiment showing that anti-TRPM6 antibody was able to recognize TRPM6, by immunoprecipitation using anti-TRPM6 antibody in GFP-TRPM6 transfected HEK cells (**D**). Western blot analysis of TRPM6 immunoprecipitated from WT and KD mouse T cells and kidneys. Full gel images are provided in Supplementary Fig. S6.

We next performed Western blot analysis using anti-TRPM7 and anti-TRPM6 antibodies to determine if these proteins were expressed in T cells. Both channels have identical I-V relations, are Mg^2+^-sensitive, and both possess functional eEF2K-like kinase domains^13,16,60^. Thus, TRPM6 and TRPM7 could potentially substitute for each other’s function in cells where both are expressed. TRPM7 protein was highly expressed in PMA/ionomycin and anti-CD3/CD28 stimulated T cells, but we were unable to detect significant TRPM7 protein bands in resting T cells (Fig. 5A). As expected, TRPM7 kinase in resting WT T cells was able to phosphorylate MBP, but no MBP phosphorylation was detected in KD mutant cells (Fig. 5B)^10^.

We tested the anti-TRPM6 antibody in control experiments with mouse kidney and human TRPM6 transfected HEK293 cells: in both cases bands corresponding to TRPM6 were detected (Fig. 5C,D). Only a faint band was detected in resting T cells from KD mutant mouse, however. No TRPM6-specific bands were detected in PMA/ionomycin stimulated WT and KD mouse T cells (data not shown). These results suggest that TRPM6 protein levels in T cells may be very low, beyond the detection limit of our method. Expression of TRPM6 mRNA was previously reported in human peripheral blood lymphocytes and murine splenic T lymphocytes^57^, however TRPM6 protein expression in T cells and its functional significance will require further investigation.

### TRPM7 channel activity in murine splenic T cells

**Figure 6:**
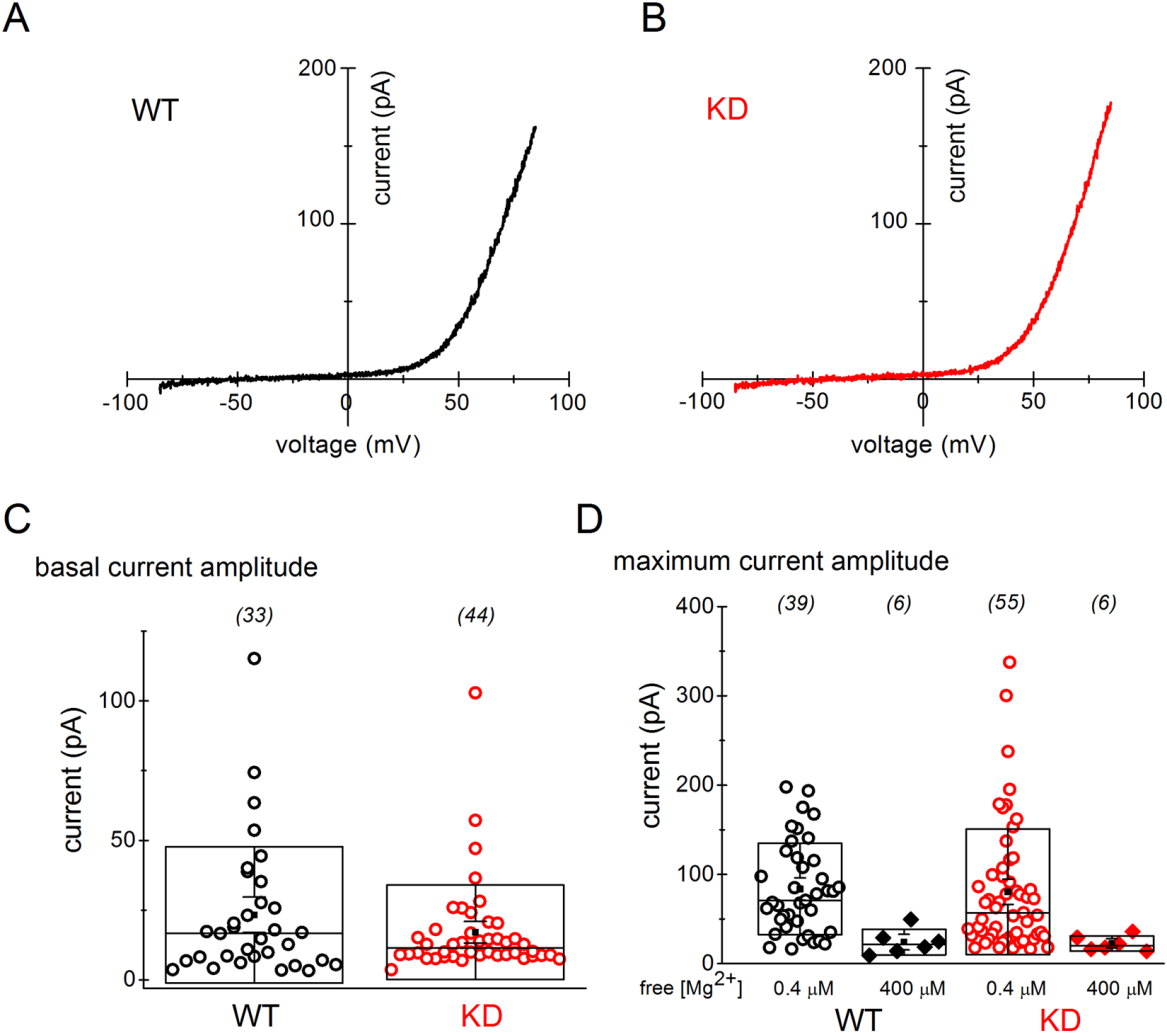
Electrophysiological characterization of T-cell TRPM7 channel activity. (**A**,**B**) TRPM7 current-voltage relations obtained from WT and KD mouse splenic resting T cells in whole-cell patch clamp. (**C**) Basal TRPM7 current amplitudes measured at break-in in WT and KD mutant T cells. (**D**) Maximum current amplitudes in WT and KD mutant T cells with 400 nM (circles) and 400 µM (diamonds) calculated free Mg^2+^ in the pipette. Numbers of cells in each data set are shown in parentheses. Data are from 11 WT and 14 KD mice. Differences between WT and KD T-cell basal (**C**) and maximum (**D**) TRPM7 currents were not statistically significant (Student’s t-test).

To evaluate its function, we recorded TRPM7 channel currents in cells isolated from WT and KD mutant mice. Figure 6A,B shows I-V plots for WT (black) and KD (red) T cells. In both cell types TRPM7 showed the characteristic steep outward rectification with comparable current amplitudes. Basal TRPM7 current amplitudes were also similar (Fig. 6C). Maximum current amplitudes were not different in WT and KD mouse splenic T cells (Fig. 6D). In order to confirm the identity of the channels we recorded, we included 400 µM free Mg^2+^ in the internal solution, which resulted in a substantial reduction in maximum currents, as expected for TRPM7 channels and in agreement with our findings in murine macrophages^6,10^. It should be noted that under this experimental paradigm, TRPM6 channels would also be detected and may have contributed to our measurements.

### Store-operated Ca^2+^ entry in WT and TRPM7 KD mutant splenocytes

Persistent Ca^2+^ elevations are necessary for efficient blastogenesis and proliferation of T lymphocytes. Because calcium is required for activation of NFAT transcription factor via calcineurin, a Ca^2+^-dependent phosphatase^61^, we reasoned that a calcium signaling defect in the KD mutant mouse splenocytes might be responsible for impaired blastogenesis and proliferation. The main source of Ca^2+^ in human and murine lymphocytes is Ca^2+^ influx through store-operated Orai channels^62^.

**Figure 7:**
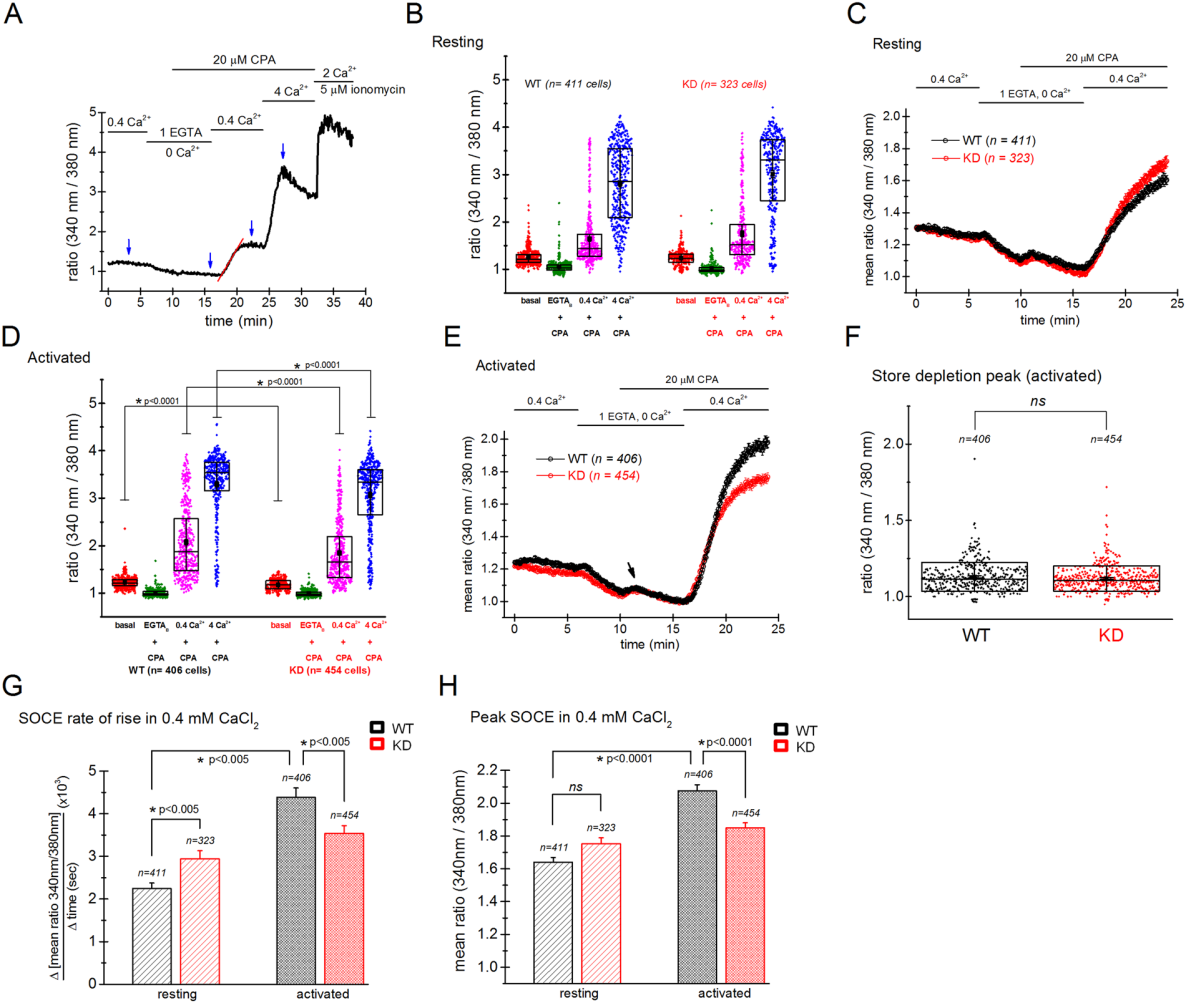
Store-operated calcium entry is reduced in activated KD splenocytes. (**A**) The experimental paradigm for measuring SOCE in Fura-2 loaded splenocytes. Arrows indicate time points where the measurements were taken for further analysis. The slope measured to determine the SOCE rise time is shown as red line. (**B**,**D**) Basal Ca^2+^ levels, Ca^2+^ levels immediately after CPA-induced store depletion and SOCE amplitudes in 0.4 mM and 4 mM Ca^2^ are shown in resting and PMA/ionomycin-activated WT and KD splenocytes, respectively. (**C**,**E**) The mean SOCE in 0.4 mM external Ca^2+^ in resting and activated WT and KD splenocytes with respect to time. Time courses represent mean responses from the same cells plotted in (**B** and **D**), respectively. Arrow shows CPA-induced store depletion transient. (**F**) Store depletion peaks in CPA (measured at  black arrow in E) were not significantly different (at p = 0.05). (**G**) SOCE rate of rise (initial slope) measured in 0.4 mM external Ca^2+^ in resting and activated WT (black) and KD (red) splenocytes. (**H**) The mean SOCE amplitudes in 0.4 mM external Ca^2+^ in resting and activated WT (black) and KD (red) splenocytes. Splenocytes were activated with PMA/ionomycin for 24 hours. Data collected from 5 WT and 5 KD mice.

Since SOCE has been well documented to stimulate T-cell proliferation^37^, we examined SOCE in WT and KD mutant splenocytes. We employed the Ca^2+^ re-addition protocol^63^ to specifically activate SOCE. Ca^2+^-dependence of proliferation becomes steeper above 0.1 mM (Fig. 2E,G), we therefore measured SOCE at 0.4 mM and 4 mM Ca^2+^. The bathing solutions contained 0.4 mM Mg^2+^ to approximate the concentration of Mg^2+^ present in RPMI culture medium. The experimental paradigm is shown in Fig. 6A where SOCE was evoked in resting and activated splenocytes by cyclopiazonic acid (CPA)-induced Ca^2+^ store depletion and subsequent reintroduction of external Ca^2+^. We found that basal calcium levels or SOCE were not significantly different in WT and KD resting splenocytes (Fig. 7B,C). Interestingly, the initial slope of Ca^2+^ rise in 0.4 mM Ca^2+^, used as a measure of Ca^2+^-release activated Ca^2+^ (CRAC) channel activity (e.g.^58,64)^ was slightly higher in resting KD mutant cells (Fig. 7G). However, after 24 hours of PMA/ionomycin stimulation, basal calcium levels and SOCE in 0.4 and 4 mM Ca^2+^ were consistently lower in KD mutant cells compared to WT (Fig. 7D,E,H). The mean peak SOCE ratio in activated WT cells was 2.076 ± 0.037 whereas for KD it was 1.85 ± 0.031. SOCE rate of rise in KD mutant splenocytes was 0.00354 ± 1.83011E-4 compared to 0.00437 ± 2.21264E-4 in WT, a 19.2% reduction (Fig. 7G). By contrast, the store Ca^2+^ content of WT and KD mutant splenocytes was the same (Fig. 7F). However, it should be noted that as CPA reveals a relatively slow sustained leak of Ca^2+^ from the stores, the store depletion peak amplitude could be underestimated due to cellular Ca^2+^ buffering and Ca^2+^ extrusion. In PMA/ionomycin treated WT splenocytes, SOCE rate of rise was increased approximately twofold compared to resting cells (Fig. 7G). In control experiments the cells were pre-incubated in 1 µM 3,5-bistrifluoromethyl pyrazole 2 (BTP2) (YM-58483), a SOCE inhibitor^65,66^ which abolished SOCE in the majority of cells (data not shown), confirming that Ca^2+^ elevations recorded in our measurements were indeed store-dependent. We concluded from these experiments that PMA/ionomycin-induced activation increases the expression of SOCE, as shown in Fig. 7. This up-regulation of SOCE upon activation is impaired by TRPM7 kinase inactivation, which is particularly apparent when comparing SOCE rates of rise (Fig. 7G). Thus, reduced blastogenesis and proliferation in KD T cells may in part be explained by diminished SOCE.

### Reduced blastogenesis in TRPM7 KD mice upon stimulation with anti-CD3/CD28

**Figure 8:**
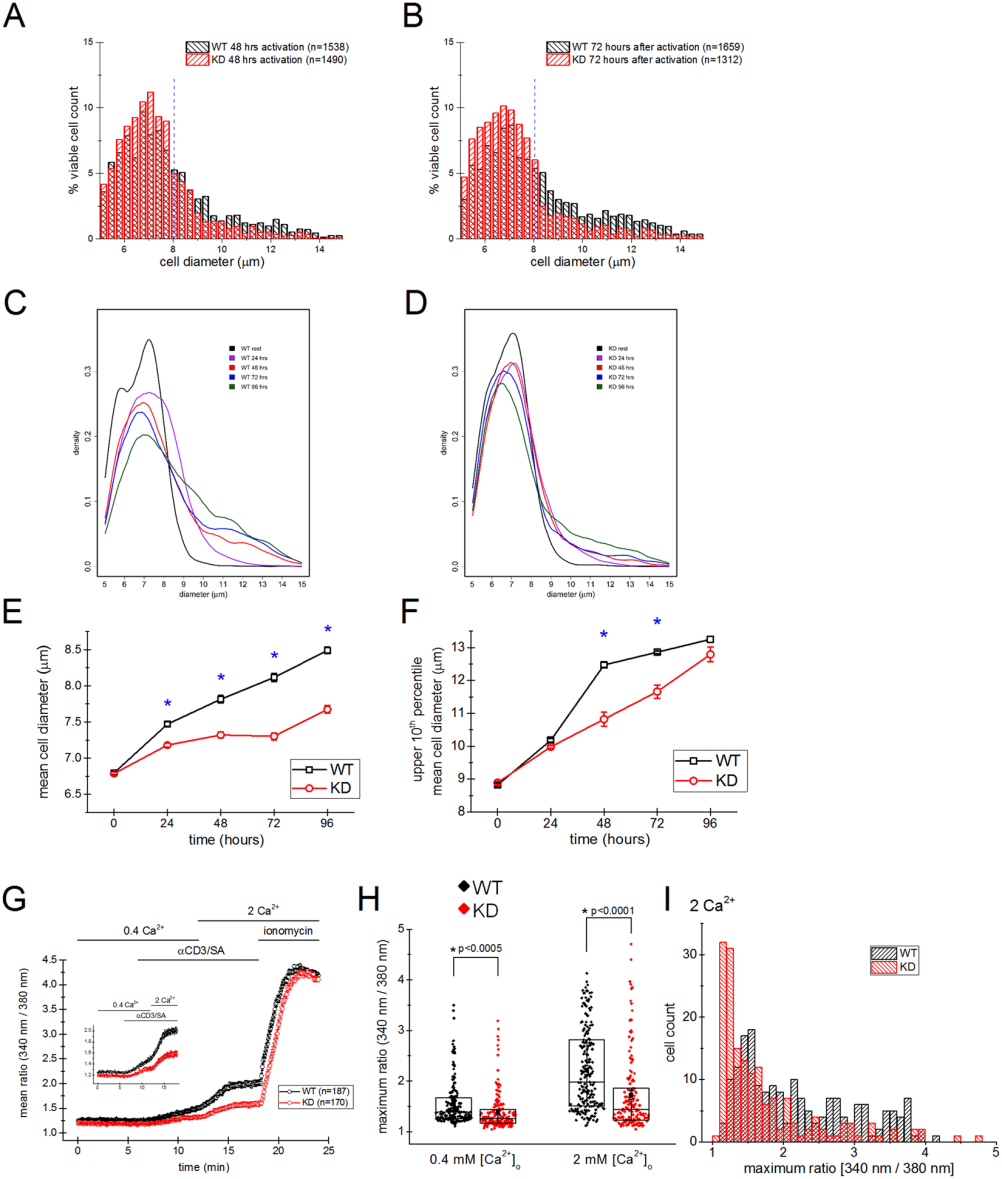
Blastogenic response to anti-CD3/anti-CD28 antibodies and Ca^2+^ elevations in response to anti-CD3 crosslinking. (**A**,**B**) Histograms showing the distribution of WT and KD T-cell diameters 48 and 72 hrs after activation with anti-CD3/CD28 antibody coated beads. The dotted line is at 8.05 µm. (**C**,**D**) Average shifted histograms showing the blastogenic response 24, 48, 72 and 96 hrs after activation of WT and KD T cells. (**E**,**F**) The mean diameter of all cells combined and upper 10^th^ percentile of cells with larger diameter at rest, 24, 48, 72 and 96 hrs after activation, respectively for data shown in (**C** and **D**). (**A**–**F**) Activation was performed with anti-CD3/anti-CD28 coated beads at 1:1 bead-to-cell ratio. Data in A-F were collected from 3 WT and 3 KD mice (*denotes p < 0.0001). (**G**) Time courses of Fura-2 dye mean fluorescence ratios in response to biotinylated anti-CD3 (αCD3) crosslinking by streptavidin (SA) in 0.4 and 2 mM [Ca^2+^_o_] followed by ionomycin control. Inset shows time courses in 0.4 and 2 mM Ca^2+^ without the ionomycin step. (**H**) Maximum Ca^2+^ response to anti-CD3 crosslinking in individual cells in (**G**) in 0.4 and 2 mM [Ca^2+^]_o_. (**I**) Distribution of maximum Ca^2+^ responses in 2 mM [Ca^2+^]_o_ generated from points shown in (**H**). Data in (**G**–**I**) were collected from 3 WT (187 cells) and 3 KD mutant (170 cells) mice.

PMA activates protein kinase C (PKC) enzymes, mainly the PKCθ subtype in lymphocytes^67–70^, whereas ionomycin is a Ca^2+^ ionophore that increases cytosolic Ca^2+^ ^71^. Thus, PMA and ionomycin bypass the early steps of T-cell receptor mediated processes during lymphocyte activation. Therefore, any effect of TRPM7 kinase inactivation on the TCR signaling cascade upstream of PKC activation would not be identified by activating T cells with PMA/ionomycin. To determine if the effect of TRPM7 kinase deficiency on T-cell activation occurred with more physiological activation stimuli mediated through TCR, we measured blastogenesis in T cells activated with anti-CD3/anti-CD28 antibody coated beads. We observed reduced blastogenesis of KD T cells upon simulation with anti-CD3/anti-CD28 coated beads compared to WT at 24, 48, 72 and 96 hours of activation (Fig. 8A–F, Supplementary Fig. S5), consistent with what was observed with PMA/ionomycin stimulation (Fig. 3). The mean diameters of all activated T cells combined were significantly reduced in KD compared to WT mice (Fig. 8E). To investigate whether there were any significant differences in the upper tails of the distributions, we specifically looked at the largest 10% of cells. The mean diameter of upper 10^th^ percentile of cells was significantly lower in KD compared to WT at 48 and 72 hours of anti-CD3/CD28 stimulation (Fig. 8F). For WT and KD resting T cells, upper 10% were above 8.05 µm in diameter. Therefore, we calculated the percentage of cells above and below 8.05 µm diameter at different times of activation. The percentage of cells below 8.05 µm was higher in KD, whereas the percentage of cells above 8.05 µm was lower (Supplementary Fig. S5). These results suggest that the percentage of blasting T cells as well as mean extent of enlargement were reduced in KD mice, indicating that TRPM7 kinase plays an important role in TCR-mediated activation process.

### Ca^2+^ responses to anti-CD3 antibody stimulation are reduced in KD mutant T lymphocytes

In order to investigate if Ca^2+^ elevations induced by TCR ligation differed in WT and KD mutant T cells, we acutely applied streptavidin to crosslink biotin-conjugated anti-CD3 antibodies bound to the surface of purified T cells^72^. As expected, streptavidin application in 0.4 Ca^2+^_o_ resulted in a Ca^2+^ rise (Fig. 8G). Increasing [Ca^2+^]_o_ to 2 mM resulted in higher Ca^2+^ in individual cells, showing that Ca^2+^ influx is the major contributor to the overall response. Ca^2+^ elevations were significantly smaller in KD mutant T cells compared to WT at both 0.4 and 2 mM [Ca^2+^]. Basal Ca^2+^ levels were also lower in KD (Fig. 8G). Most likely, the basal Ca^2+^ levels in WT were increased by short stimulation with anti-CD3 before streptavidin was added (see *Materials and Methods*). The marked difference between mean cytosolic Ca^2+^ levels in WT and KD T cells in 2 mM [Ca^2+^]_o_ (22.4%) was due to a reduction in the number of high responder cells (Fig. 8H,I). The mean percentages of cells responding to anti-CD3/streptavidin also tended to be higher in WT (65.1%) compared to KD (41.4%). We did not observe Ca^2+^ oscillations which have been reported under similar experimental conditions in human lymphocytes^62^. In a recent study, a slightly reduced Ca^2+^ elevation in response to anti-CD3/CD28 stimulation was reported in T cells of TRPM7 KD mutant mice^73^.

### Expression of cytokines in activated T cells

**Figure 9:**
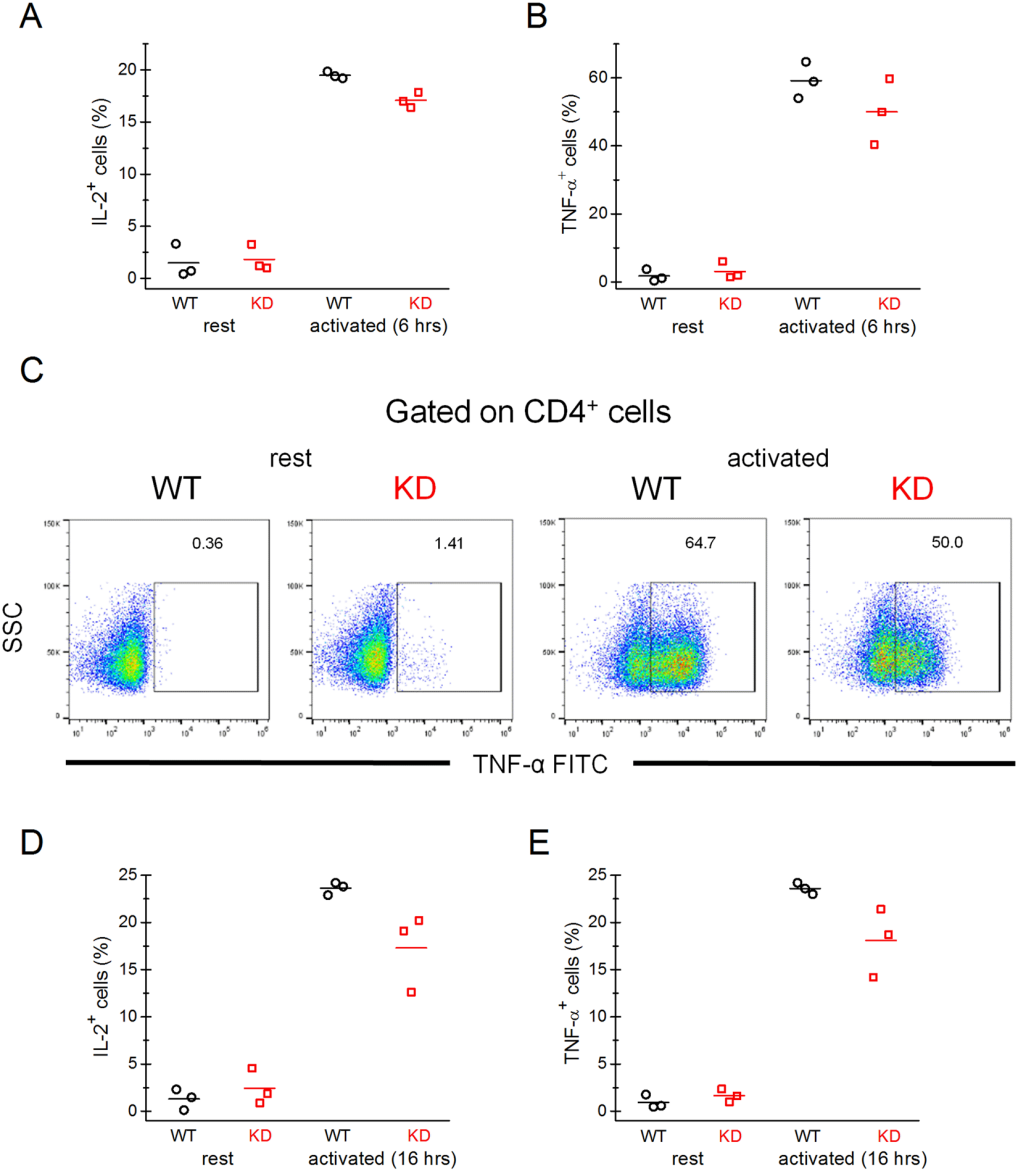
Expression of IL-2 and TNF-α cytokines in activated KD CD4^+^ T cells is reduced. (**A**,**B**) Expression of IL-2 and TNF-α in WT and KD CD4^+^ splenic T cells, left unstimulated or stimulated for 6 hours with PMA/ionomycin, in the presence of Brefeldin A during the last 5 hrs, determined by flow cytometry. (**C**) Representative flow plots from one independent experiment for data shown in (**B**). (**D**,**E**) IL-2 and TNF-α expression in resting cells and 16 hrs after PMA/ionomycin stimulation of CD4^+^ T cells. Brefeldin A was present during the last 6 hrs. For (**A**-**E**), data were collected from three independent experiments performed on samples from 3 WT and 3 KD mice. Horizontal lines represent arithmetic means. The p value for the differences in the percentage of IL-2^+^ and TNF-α^+^ cells in WT and KD CD4^+^ T cells in activated conditions were p = 0.007 (**A**), p = 0.225 (**B**), 0.058 (**D**) and 0.061 (**E**), as determined by Student’s t-test. For resting T cells, the differences between WT and KD in A, B, D and E were not statistically significant.

Using flow cytometry, we investigated the expression of cytokines IL-2 and TNF-α in WT and KD CD4^+^ T cells upon stimulation. Consistent with the proliferation data (Fig. 2A), after stimulation with PMA/ionomycin for 6 hours, KD T cells showed a modest but significant reduction in IL-2 expression (Fig. 9A). In three independent experiments, the expression of TNF-α was reduced by about 24% in KD CD4^+^ T cells in two experiments but not in the third one (Fig. 9B,C). 16 hours after activation, both IL-2 and TNF-α production was lower in KD compared to WT, with differences not reaching statistical significance, however (p = 0.058 and 0.061 for IL-2 and TNF-α, respectively) (Fig. 9D,E). Collectively, the data suggest that TRPM7 kinase activity may potentially be involved in the expression of IL-2 and TNF-α upon stimulation of CD4^+^ T cells.

## Discussion

Here, we have investigated the consequences of global genetic inactivation of TRPM7 kinase in the mouse spleen. We used the previously characterized TRPM7 K1646R mutant mouse to this end^10^. The function of TRPM7 kinase can be successfully studied in this mouse model as the animals are born normally and have a normal lifespan, unlike mice where the full TRPM7 gene or the kinase domain has been deleted, resulting in embryonic lethality^2,10,29^. We describe a proliferative defect in splenic T cells of the KD mutant mouse. For evaluating the T-cell proliferative competence we used two assays: single-cell diameter measurements with a cell viability analyzer and a plate reader based colorimetric proliferation assay. In T cells stimulated with PMA/ionomycin, blastogenesis and proliferation were significantly impaired in KD compared to WT, in addition to decreased expression of cytokines by KD CD4^+^ T cells (Figs 2, 3 and 9). Stimulation with anti-CD3 and anti-CD28 antibodies also resulted in reduced blastogenesis of KD T cells (Fig. 8A–F).

Inactivation of TRPM7 kinase increased somewhat the sensitivity of T cell proliferation to rapamycin, CsA and and FK506 (Fig. 2B–D and Supplementary Fig. S1). These results suggest that both Ca^2+^ dependent and independent pathways of T cell activation are slightly defective in the KD T cells. Since TCR signaling steps are bypassed during PMA/ionomycin activation, KD mutant mouse T cells potentially have a defect downstream of TCR. Interestingly, full TRPM7 gene deletion in DT40 avian pre-B lymphocytes was found to impair activation at or upstream of mTOR complex 1 signaling, but this was explained by TRPM7 ion transport function rather than the kinase^74^.

Since TRPM7 channels were previously reported to conduct Ca^2+^, Mg^2+^ as well as other divalent cations, we tested the dependence of T-cell proliferation on the concentrations of these ions. The RPMI medium, widely used for culturing T lymphocytes, usually contains approximately 0.4 mM Ca^2+^ and 0.4 mM Mg^2+^. We therefore tested the effects of one metal cation on T-cell proliferation when the other was kept at a constant concentration (Fig. 2). We found that both Ca^2+^ and Mg^2+^ stimulate proliferation, as reported previously (e.g.^75–77^). Ca^2+^-dependence of proliferation was much steeper than its dependence on Mg^2+^. Lowering Ca^2+^ and Mg^2+^ in the culture medium allowed us to readily detect differences in proliferation between the WT and KD mice. The largest differences were seen after only 24 hrs of stimulation: at all tested divalent concentrations the KD mutant cells were proliferating less robustly than their WT counterparts. At 48 hrs this difference was smaller and disappeared altogether at higher Ca^2+^/Mg^2+^ concentrations ([Ca^2+^]_o_ >170 µM and [Mg^2+^]_o_ >50 µM). We did not investigate the detailed consequences of changing extracellular Ca^2+^/Mg^2+^ concentrations on cytosolic Ca^2+^ and Mg^2+^ homeostasis in T cells, which may shed more light on the mechanism by which these cations affect proliferation. In activated human T cells, increasing external Ca^2+^ from 50 to 300 µM only resulted in modest increases in cytosolic Ca^2+^ ^78^.

In DT40 pre-B cell line, full TRPM7 deletion resulted in Mg^2+^-dependent reductions in cell size and proliferation. These defects could be reversed by media supplementation with Mg^2+^, implicating the channel function of TRPM7, however, intracellular free Mg^2+^ did not undergo corresponding changes^74^. In some instances at least, changing external [Mg^2+^] acutely may not result in significant changes in global cytoplasmic Mg^2+^(see^46,79^). SOCE is required for efficient T-cell proliferation^36,47^. A point mutation in the store-operated Ca^2+^ channel pore subunit Orai1 causes severe combined immunodeficiency (SCID) syndrome^80^. Knockout of the ER Ca^2+^ sensors STIM1 and STIM2 in mice resulted in splenomegaly and reductions in proliferation, cytokine secretion and NFAT nuclear translocation in T cells upon TCR engagement^81^. We, therefore, hypothesized that reduced blastogenesis and proliferation in KD mutant T cells may arise from suppressed SOCE. This was indeed the case (Fig. 7): SOCE in PMA/ionomycin treated splenocytes was consistently lower in KD mutant compared to WT. Measurements of the initial slope of Ca^2+^ rise in low bathing Ca^2+^ (0.4 mM) suggested that the reduction is likely due to CRAC channels themselves. A recent publication has suggested that TRPM7 kinase modulates SOCE in DT40 cell line potentially by interacting with STIM proteins^82^, supporting our findings in primary murine cells.

Upon activation of WT T cells, the Ca^2+^ EC_50_ decreased from 175 µM to 130 µM (Fig. 2). This shift in EC_50_ (Ca^2+^) was less prominent in KD mutant (a decrease from 159 µM to 152 µM). We also observed an upregulation of SOCE in activated WT splenocytes compared to resting WT cells, which was abolished in KD splenocytes (Fig. 7E,G,H). This suggests that the leftward shift of EC_50_ (Ca^2+^) in activated WT cells could be mediated by upregulation of SOCE during PMA/ionomycin stimulation which is missing in the KD mouse. Thus, TRPM7 kinase may potentially mediate the process of SOCE upregulation during T-cell activation. SOCE upregulation reported here is in agreement with our previous findings in human T cells (Fig. 7^58^). Alternative possibilities include reduced K^+^ conductances (e.g. K_V_1.3 and K_Ca_3.1) which would result in a more depolarized membrane potential and reduced driving force for Ca^2+^ entry, corresponding to diminished SOCE^62^. Since TRPM7 is a ubiquitous protein, likely all K_V_1.3 and K_Ca_3.1 studies have been done in the presence of intact kinase and it is therefore not known if these channels are regulated by TRPM7 kinase. T cells also express constitutively active K^+^ channels of the two-pore family^83–85^ which could also be regulated by TRPM7 kinase. Ca^2+^ elevation due to ionomycin depends upon the membrane potential^86^. Thus, a hyperpolarized membrane potential in low extracellular K^+^ increased Ca^2+^ influx through ionomycin-induced pathway whereas a depolarized membrane potential in high K^+^ decreased it (Supplementary Fig. S4)^86^. Therefore, depolarization of KD mutant cells would be expected to suppress blasting and proliferation under our stimulation conditions. Interestingly, in T cells obtained from SCID patients with defective SOCE, the voltage sensitivity of K_V_1.3 and its activation kinetics were altered^80^. Reduced SOCE in KD splenocytes may therefore reflect reduced K^+^ conductance in addition to reduced expression or altered properties of SOCE. Stimulation of purified splenic T lymphocytes with anti-CD3 crosslinking generated large Ca^2+^ elevations (Fig. 8G–I). These elevations arise primarily from Ca^2+^ release from ER stores by inositol trisphosphate generation and Ca^2+^ influx through CRAC channels^37,62^. In KD mutant cells Ca^2+^ elevations were significantly diminished (Fig. 8). Based on our data showing no difference in store content between WT and KD mutant splenocytes (Fig. 7F), we propose that Ca^2+^ influx was reduced in the mutant. In agreement with this, Ca^2+^ rises in 2 mM were larger than in 0.4 mM Ca^2+^, and were diminished in the KD mutant cells to a greater degree (Fig. 8H). It should be kept in mind that in the presence of external Ca^2+^, store release transients are masked by influx.

Mean SOCE amplitudes in resting cells from KD mice were larger than in WT even though this difference was not statistically significant (Fig. 7B,C,H). The initial slope of SOCE rise, on the other hand, was significantly increased (Fig. 7G), suggesting that the Ca^2+^ influx through Orai channels is potentiated. Thus, TRPM7 kinase inactivation potentiated Ca^2+^ influx in resting cells while decreasing it in activated cells. Divergent regulation of SOCE in resting cells may reflect differences in the predominant Orai isoform mediating SOCE in these cells^62^. Resting and activated lymphocytes differ fundamentally in their energy metabolism and may also differ in their complement of Ca^2+^ signaling molecules^87,88^. The observation that the increase in SOCE amplitude does not reach statistical significance may be a reflection of homeostatic regulation of Ca^2+^ by the plasma membrane Ca^2+^ pump, which is more active in resting cells, or by mitochondria^62,88^. The precise function of SOCE in quiescent T lymphocytes is not well understood.

Similar to Ca^2+^, we found that EC_50_ for Mg^2+^ also shifts leftwards at 48 hours in WT T cells (i.e. decreases from 65 to 39 µM) in proliferation assay (Fig. 2). The slope decreased from 2.6 ± 0.8 to 1.1 ± 0.1. This change was less prominent in KD T cells where EC_50_ decreased from 63 µM to 52 µM and the slope decreased from 2.5 ± 0.4 to 1.5 ± 0.1, suggesting that a Mg^2+^ transport pathway may be upregulated in T cells during activation in WT but not in KD mutant. The KD mice display normal blood serum Ca^2+^ and Mg^2+^ levels and the intracellular concentrations of these cations in platelets were also normal^10,89^. Thus the effects we observed in KD T-cell proliferation under reduced [Ca^2+^]_o_ and [Mg^2+^]_o_ (Figs 2 and 3) are unlikely to result from cellular deficiency of these cations in resting cells.

We found that expression of TRPM7 protein is increased in both WT and KD mutant T cells activated with PMA/ionomycin or anti-CD3/CD28 antibodies (Fig. 5). However, we found no evidence for transcriptional regulation of TRPM7 or TRPM6 (Fig. 4) under the same conditions. TRPM7 mRNA expression levels did not noticeably change in the presence calcineurin inhibitors CsA and FK506, suggesting that NFAT does not regulate the expression of TRPM7 mRNA. This is in agreement with^60^ which did not identify NFAT binding sites in the TRPM7 promoter region. Collectively, these data suggest that TRPM7 protein expression is upregulated upon T-cell activation at the level of translation or protein degradation and its kinase activity is not required for this process. Potential mechanisms of post-transcriptional regulation include TRPM7 mRNA 5′-leader sequence, which contains two upstream open reading frames that regulate translation initiation in a Mg^2+^-dependent manner and TRPM7 protein degradation through activation of calcium-dependent cysteine proteases, calpains^90,91^.

TRPM7 channel activity in KD mutant T cells was similar to that in WT, underscoring the dispensability of the kinase function for ion conduction (Fig. 6)^10,23^. Both Ca^2+^ and Mg^2+^ have been reported to conduct inward current through overexpressed TRPM7 channels, however, these patch-clamp recordings were performed at high external Mg^2+^ concentrations of 10–120 mM and at very negative membrane potentials^43^. In view of apparently unchanged TRPM7 channel properties in KD mutant cells (Fig. 6), it is unlikely that the proliferative defect we observed under lower [Ca^2+^]_o_ and [Mg^2+^]_o_ (Figs 2 and 3) is related to TRPM7 ion conduction pathway. Additionally, at external Mg^2+^ concentrations of 0.4 mM and below, influx of Mg^2+^ into lymphocytes through TRPM7 would be relatively modest, assuming a T-cell membrane potential of ~−50 mV^92–94^ and ~1 mM [Mg^2+^]_i_^11^. The possibility exists, therefore, that extracellular Ca^2+^ and Mg^2+^ stimulate blastogenesis and proliferation by acting on receptors coupled to intracellular signaling cascades, such as the Ca^2+^ sensing receptor (CaSR) which can be activated by Ca^2+^ and Mg^2+^ from outside^95,96^.

We also found that the spleens of KD animals are enlarged. Splenomegaly could be due to mild diffuse extramedullary hematopoiesis (EMH) with accumulation of erythroid, myeloid and megakaryocytic precursors, as determined by histological evaluation (Fig. 1). White blood cell differentials and complete blood counts were, however, normal. We also noticed that the KD spleens were of darker red color than WT (see Fig. 1A). Recently, mice with conditional deletion of TRPM7 in megakaryocytes and platelets were characterized^89^. These mice did not exhibit splenomegaly but showed expansion of red pulp with increased number of megakaryocytes in the spleen^89^. The darker color (but not enlargement) of spleens in the KD mice may possibly be explained by a mild defect in thrombopoiesis in addition to EMH.

Deletion of full TRPM7 selectively in T cell lineage was reported to impair thymopoiesis with the accumulation of T cells at double-negative (DN) stage. Splenomegaly was not observed in these mice at young age but a small decrease in the density of T cells in the spleen and lymph nodes occurred^2^. We have not observed any defects in thymopoiesis with accumulation of thymic T cells at DN stage in KD mice (Fig. 1C). The spleen and lymph nodes of KD mice also showed no significant differences in the percentage of lymphocyte subsets compared to WT (Fig. 1C,D). These results suggest that the defects observed by Jin *et al*.^2^ may primarily be due to abolished TRPM7 channel and not kinase function.

TRPM7 channel activity is required for Fas-receptor-induced apoptosis of T cells. Thus Fas-mediated apoptosis could potentially be altered in KD mice as the autophosphorylation of Ser1511, which we originally identified^23^, could influence the involvement of TRPM7 during this process^27^. Fas-deficient C57BL/6-*lpr*/*lpr* mice exhibit splenomegaly with increased EMH, which further supports this possibility^97^. Our diameter measurements were performed on viable cells (Figs 3 and 8), but we saw no difference in viability between WT and KD mutant cells, (Supplementary Fig. S2) indicating that the effect of kinase inactivation on T-cell blastogenesis is unlikely to involve apoptosis. These data also show that functional TRPM7 kinase is not required for T-cell viability.

Despite impaired KD T-cell activation, the resting (unstimulated) KD T cells had larger volumes (by ~4%). Although small, this increase could in part account for the observed splenomegaly. In addition to the expression of activated state surface markers by KD T cells, the EC_50_ (Ca^2+^) value (159 µM) is lower than WT at 24 hours after activation and the resting KD splenocytes showed slightly higher SOCE rate of rise than WT (Fig. 7G). These results suggest that T cells in spleens of KD animals are in a “hyperactivated” state. Splenomegaly in association with “hyperactivated” phenotype of T cells was observed in several mouse models with deficiency of proteins involved in T-cell activation process such as STIM1-deficient, CD4^+^ T cell specific STIM1 and 2 deficient, Y136F LAT knock-in, NFAT1 and NFAT4 double knockout, IL-2 deficient and Fas deficient mice^54,55,81,97–100^. A possibility exists that KD mice exhibit a mild lymphoproliferative disease similar to global STIM1-deficient mice or mice with CD4^+^ T cell specific STIM1 deficiency^81,98^, potentially due to defective SOCE in KD T cells.

Future studies will address the cause of splenomegaly and “hyperactivated” phenotype of T cells in KD mice in addition to the mechanism through which TRPM7 kinase regulates T-cell activation process. In summary, the presented findings provide a novel insight into the physiological role of TRPM7 kinase in regulating T-cell function.

## Methods

### Transgenic mice

TRPM7 K1646R knock-in mice have been described in^10^. Briefly, homologous recombination of WT TRPM7 gene in embryonic stem cells was performed with a targeting vector containing TRPM7 allele with a point mutant encoding TRPM7 K1646R protein. The Neo cassette was deleted and the heterozygous mice were bred together to generate homozygous TRPM7 K1646R “kinase-dead” mice (referred to as KD mice). The KD mice were generated in C57BL/6 background. Experiments were performed with 1–6 months old mice of either sex unless otherwise specified.

### Complete blood count and spleen histology

Mouse blood was collected by cardiac puncture immediately after euthanasia and placed in Microvette EDTA-coated tubes (Sarstedt Inc., Newton, NC). Spleens collected from the same mice were fixed immediately in 10% neutral buffered formalin. Complete blood count with spleen processing, white blood cell differentials and histological evaluation was performed at Comparative Pathology and Mouse Phenotyping Shared Resource, Department of Veterinary Biosciences of The Ohio State University (Columbus, Ohio).

### Splenic T-cell isolation

All procedures involving mice were performed according to protocols approved by the Laboratory Animal Care and Use Committees of Wright State University, Kumamoto University and University of the Ryukyus according to the NIH guidelines. Mouse spleen dissection and T cell isolation was performed as previously described in detail^53^. Each spleen was weighed on a scale shortly after dissection. For splenocyte culture, the spleen was crushed between two glass slides to release splenocytes which were passed through 40 µm nylon cell strainer (Fisher Scientific, Fair Lawn, NJ) and cultured in RPMI-1640 (Lonza, Walkersville, MD) containing 2 mM L-glutamine, supplemented with 10% fetal bovine serum (Thermo Fisher Scientific, Waltham, MA), 1 mM 1,4-dithiothreitol (DTT; Research Products International, Mount Prospect, IL), 50 IU/ml penicillin and 50 µg/ml streptomycin (MP Biomedicals, Irvine, CA). T cells were purified from splenocytes using nylon wool fiber columns (Polysciences, Inc., Warrington, PA) and cultured in supplemented RPMI-1640. The purified T-cell preparation had approximately 42% CD4 and 35% CD8 cells as determined by flow cytometry (data not shown).

### Isolation of cells from thymus and lymph nodes

Thymus glands and lymph nodes (inguinal and axillary) were physically disrupted in ice cold supplemented RPMI-1640 and passed through 40 µm nylon cell strainer to obtain single-cell suspensions of thymocytes and lymph node cells.

### FACS analysis

The antibodies used were: anti-CD3 PE-Cy7 (145–2C11), anti-CD45R/B220 FITC (RA3-6B2), anti-CD4 PE-Cy7 or APC (RM4-5), anti-CD8a APC (53-6.7), CD62L FITC (MEL-14), CD44 PE-Cy7 (IM7), anti-TNF-α FITC (MP6-XT22) and anti-IL-2 FITC (JES6-5H4) (all from Thermo Fisher Scientific, eBioscience).

For staining with antibodies, 0.5–1 × 10^6^ cells were incubated in FACS buffer (DPBS, 1% FBS and 0.1% NaN_3_) with anti-mouse CD16/CD32 (Mouse BD Fc Block, BD Pharmingen, San Diego, CA) for at least 5 min at 4 °C. Fluorochrome-conjugated antibodies were added to the samples and incubated for 30 min at 4 °C in the dark. After washing off the primary antibody the samples were analyzed on Accuri C6 Flow Cytometer (BD Accuri Cytometers, Ann Arbor, MI). Flow data were further analyzed with FlowJo software (Treestar Software, San Carlos, CA).

For measuring the intracellular cytokine expression, after staining with cell surface antigens, the samples were fixed and permeabilized using BD Cytofix/Cytoperm Fixation and Permeabilization kit (BD Biosciences, San Jose, CA). Fluorochrome-conjugated antibodies (anti-IL-2 FITC or anti-TNF-α FITC) were then added to the samples and incubated for 1 hour at 4 °C in the dark, followed by two washing steps and data acquisition on the flow cytometer. Dead cells were excluded from the analysis by labeling with Fixable Viability Dye eFluor 660 (Thermo Fisher Scientific, eBioscience) prior to cell surface antigen staining. To determine TNF-α and IL-2 expression, T cells were activated with 0.125 µg/mL PMA and 0.25 µM ionomycin for 6 or 16 hours in the presence of 3 µg/ml Brefeldin A (Thermo Fisher Scientific, eBioscience) during the last 5 and 6 hours for 6 and 16 hr activation conditions, respectively. Unstimulated (resting) control cells were treated with Brefeldin A in a similar fashion. CD4 PE-Cy7 positive population was gated while analyzing the expression of cytokines using FlowJo.

### Proliferation assay

MTS (3-(4,5-dimethylthiazol-2-yl)-5-(3-carboxymethoxyphenyl)-2-(4-sulfophenyl)-2H-tetrazolium) reagent based colorimetric plate reader assay was performed using CellTiter 96 AQ_ueous_ one solution cell proliferation assay reagent (Promega, Madison, WI) according to manufacturer’s instructions^53,101^. Briefly, purified T cells were plated at a density of 1 × 10^5^ cells/well in a 96-well culture plate with 100 µl culture media, and activated using 125 ng/ml phorbol-12-myristate-13-acetate (PMA) (Acros Organics, Geel, Belgium) and 250 nM ionomycin (Sigma-Aldrich, St. Louis, MO), except for Supplementary Fig. S1 where PMA concentration was varied from 10 to 250 ng/ml. For Mg^2+^ and Ca^2+^ dependence experiments (Fig. 2), supplemented RPMI-1640 with 25 mM HEPES was treated with chelating resin (Chelex-100, Sigma-Aldrich) to remove divalent metal cations before adding back CaCl_2_ and MgCl_2_ at indicated concentrations, essentially as described in^76,77,102^. Experiments were repeated with three batches of Chelex-100 supplemented RPMI with similar results. After 24, 48 or 72 hours of activation (at 37 °C and 5% CO_2_), 20 µl of MTS reagent was added, cells were incubated further for 4 hours at 37 °C and absorbance measurements taken at 490 nm using Synergy H1 hybrid plate reader (Biotek, Winooski, VT). Concentration-response curves for cyclosporine A (Sigma-Aldrich), FK506 (Cayman Chemical, Ann Arbor, MI) and rapamycin (Santa Cruz Biotechnology, Santa Cruz, CA) were obtained by measuring proliferation in normal supplemented RPMI after 48 hours incubation with PMA/ionomycin and the drug. Proliferation was also measured by manually counting viable cell densities using a Neubauer hemocytometer (Supplementary Fig. S1). Data were collected from 2–4 replicates for each condition during each experiment and statistical analysis was performed using combined data from all the replicates from at least 3 WT or KD mice.

### Cell size determination

Purified T cells from WT and KD mice were plated in a 24-well plate at a density of 1 × 10^6^ cells/well in supplemented RPMI-1640. Cells were activated using 125 ng/ml PMA and 250 nM ionomycin in normal or chelex-treated RPMI, or activated with anti-CD3/CD28 antibody coated magnetic beads of 4.5 µm diameter (Dynabeads Mouse T-Activator CD3/CD28; Life technologies, Grand Island, NY) according to manufacturer’s instructions at a ratio of 1:1 bead-to-cell ratio in supplemented RPMI-1640 without DTT. The diameters of individual T cells before and after activation were measured using Vi-Cell cell viability analyzer (Beckman-Coulter, Fullerton, CA), essentially as previously described for ICR mouse cells^53^. Cell viability was quantitated using trypan blue stain exclusion. The cell diameter readings were plotted as histograms and further data analysis was performed using Microsoft Excel and OriginLab software (v. 2016: OriginLab Corporation, Northampton, MA).

### Reverse transcriptase PCR and quantitative real-time PCR

T cells and mouse brains were collected in TRIzol reagent (Life technologies) to isolate total RNA. Activated T-cell RNA was collected after 48 hours of stimulation with PMA/ionomycin or anti-CD3/anti-CD28 coated beads. RT-PCR was performed with 250 ng RNA per reaction except for Fig. 4B, where it was 200 ng, as previously described using Verso 1-Step RT-PCR Hot-Start Kit (Thermo Fisher Scientific)^6,10^ and specific primers listed in Supplementary Table S2. Quantitative real-time PCR (RT-qPCR) was performed in QuantStudio 7 Flex Real-Time PCR System (Applied Biosystems, Carlsbad, CA) using TaqMan Gene Expression Assay for TRPM7 (assay ID: Mm00457998_m1) and an endogenous reference, ribosomal protein S15 (RPS15, assay ID: Mm02342443_g1) from Applied Biosystems. PCR was performed in 20 µl reaction volume, with 50 ng RNA per reaction using TaqMan RNA-to-C_T_ 1-Step Kit (Applied Biosystems) in a 96 well plate with 3 replicates for each gene. Cycling conditions were 48 °C for 15 min, 95 °C for 10 min and 40 cycles of 95 °C for 15 sec and 60 °C for 1 min. Relative expression of TRPM7 mRNA in activated compared to resting T-cell samples was calculated using the ΔΔC_T_ method. Because in pilot RT-qPCR experiments GAPDH showed a modest upregulation upon T-cell stimulation (see also Fig. 4A), we used RPS15 as an internal control^58^.

### Kinase assay

Splenic T cells from WT or TRPM7 KD mice were lysed in lysis buffer (50 mM Tris-HCl, pH 7.5, 120 mM NaCl, 0.5 mM DTT, 1.5 mM MgCl_2_, 0.2 mM EDTA, 1% Triton X-100 supplemented with protease inhibitors) on ice for 20 min and centrifuged for 10 min at 15,000 rpm to remove insoluble material. Cell extracts were incubated overnight at 4 °C with rabbit polyclonal anti-TRPM7 antibody (AB15562; Millipore, Billerica, MA), followed by 1 hr incubation with protein A sepharose beads. Afterwards, the beads were washed in the lysis buffer three times and in the kinase buffer (50 mM HEPES, pH 7.0, 4 mM MnCl_2_, 5 mM DTT) once. Myelin basic protein (MBP) substrate at 50 µg/ml concentration was added to the immunoprecipitate suspended in the kinase buffer. To initiate the phosphotransferase reaction, 0.1 mM ATP and 5 µCi [γ-^32^P] ATP were added and the reaction proceeded for 30 min at 30 °C. Laemmli buffer was used to elute the proteins bound to the beads. The proteins were separated with SDS-PAGE and phosphate incorporation was evaluated by autoradiography.

### Western blotting

To detect TRPM7 and TRPM6, proteins were concentrated by immunoprecipitation using respective antibodies. Mouse kidneys were lysed by homogenization in lysis buffer. Extracts of T cells, mouse embryonic fibroblasts (MEF) or HEK293 cells (untransfected or heterologously expressing GFP tagged human TRPM6 in pEGFP-C1 vector) were obtained as described above for the TRPM7 kinase assay and incubated with anti-TRPM7 antibody (1:500) or anti-TRPM6 antibody (1:500, ACC-046; Alomone labs, Israel) overnight at 4 °C, followed by incubation with protein A sepharose beads for 1 hr. Subsequently, the beads were washed three times in the lysis buffer. Proteins bound to the beads were eluted in Laemmli buffer, subjected to SDS-PAGE and transferred to PVDF membrane (Millipore). Membranes were blocked for non-specific binding in Blocking One (Nacalai Tesque, Kyoto, Japan) and incubated with anti-TRPM7 or anti-TRPM6 antibody overnight at 4 °C. The membranes were washed afterwards and incubated for 1 hr with HRP-conjugated anti-rabbit antibody (1:2000; Dako, Carpenteria, CA). After washing, membranes were incubated with Amersham ECL Prime (GE Healthcare, Piscataway, NJ) and the immunoreactive proteins visualized with ImageQuart400 (GE Healthcare).

### Patch-clamp electrophysiology

TRPM7 currents were recorded using whole-cell patch clamp in murine splenic T cells as described previously for murine macrophages^10^. Briefly, recordings were obtained using HEKA EPC10 patch-clamp amplifier (HEKA Elektronik, Lambrecht, Germany) from T cells that were either isolated on the day of the experiment or cultured for 1–3 days in supplemented RPMI medium. Pipettes were manufactured from borosilicate capillary glass (Harvard Apparatus, Holliston, MA) on a Flaming-Brown puller (Sutter Instrument Company, Novato, CA). Internal (pipette) solution contained 112 mM glutamic acid, 10 mM HEDTA, 60 µM MgCl_2_, 8 mM NaCl, 5 mM CsF, 10 mM HEPES, pH 7.3 (adjusted with CsOH), yielding 400 nM calculated free [Mg^2+^]. External (bathing) solution contained 2 mM CaCl_2_, 4.5 mM KCl, 3 mM CsCl, 140 mM NaAspartate, 10 HEPES-Na^+^, 0.5 mM glucose, pH 7.3. TRPM7 channel currents were evoked by applying voltage ramps from −85 to +85 mV every 2.5 seconds. I-V relations and current amplitudes at 83.4 mV in WT and KD T cells were collected and plotted for comparison (Fig. 6). A minority of cells never developed TRPM7 currents during several minutes of recording with low internal Mg^2+^ and were excluded from analysis. All experiments were performed at room temperature (23–25 °C). HEPES-Na^+^ and HEDTA were from Acros Organics. All other salts were purchased from Sigma-Aldrich.

### Fluorescence ratiometric Ca^2+^ imaging

For intracellular Ca^2+^ imaging, cells were seeded on poly-D-lysine coated 35 mm glass-bottom imaging chambers with ~1 ml solution volume. Cells were loaded with Fura-2AM calcium indicator dye (Thermo Fisher Scientific) in the recording solution containing 0.4 mM CaCl_2_ for 60 min at 37 °C. The dye was washed off afterwards and the cells remained at 37 °C for an additional 15 min to allow for full de-esterification of the dye. The recording solutions were composed of 0.4/2/4 mM CaCl_2_, 0.4 mM MgCl_2_, 140 mM NaCl, 4 mM KCl, 10 mM HEPES-Na^+^, 10 mM D-glucose, pH 7.3, ~300 mOsm. Ca^2+^-free external solution contained 1 mM EGTA, 140 mM NaCl, 4 mM KCl, 10 mM HEPES-Na^+^, 10 mM D-glucose, pH 7.3, ~300 mOsm. CPA (Sigma-Aldrich), at 20 µM concentration, was used to inhibit sarcoplasmic endoplasmic reticulum Ca^2+^ ATPase (SERCA) pumps and deplete the ER Ca^2+^ stores. For stimulation of TCR complex, splenic T cells (on the day of isolation or after overnight incubation in complete RPMI medium) were loaded with the dye and were pretreated with 5 µg/ml biotin conjugated anti-CD3 monoclonal antibody (clone 145-2C11; eBioscience, San Diego, CA). After incubating 15 min with anti-CD3 at 23–25 °C, cells were washed and transferred to the microscope stage for imaging. Streptavidin (Pierce, Thermo Fisher Scientific) at 10 µg/ml concentration was perfused at indicated times (Fig. 8) in 0.4 and 2 mM [Ca^2+^] to crosslink anti-CD3, essentially as described in^72,103^. Anti-CD3 stimulated T cells exhibiting very high baseline fluorescence ratios (≥2) prior to streptavidin application were excluded from analysis. 5 µM ionomycin (Calbiochem, San Diego, CA) was added at the end of each experiment to determine the maximal Ca^2+^ response for each cell and only cells responding to ionomycin were included in the analysis. Syringe-driven perfusion system was used to exchange solutions in the glass-bottom imaging chamber placed on the stage of an inverted microscope. Individual cells in the imaging field were illuminated every 5 seconds at 340 and 380 nm wavelengths using a Lambda 10B shutter and filter wheel (Sutter Instrument). The fluorescence was measured at 535 nm and emission ratios plotted against time. 175 W Xenon lamp was used as the light source (Intracellular Imaging, Cincinnati, OH). Images were captured with Pixelfly CCD camera (PCO Imaging, Kelheim, Germany) and InCyt Im2 software (Intracellular Imaging). For Ca^2+^ measurements, background-subtracted emitted light intensities from individual cells were averaged and plotted against time using OriginLab software.

### Statistical analysis

Results are presented as mean ± SEM and all statistical differences were determined with Student’s t-test unless otherwise specified. Vi-Cell data (Figs 3A,D, 8E and Supplementary Fig. S3) were compared using Welch’s t-test and in Fig. 8F repeated measures ANOVA was performed. Data analysis was performed using OriginLab, GraphPad and SAS (v. 9.4) software. Average shifted histograms were plotted using RStudio (v. 1.0.136).

## Electronic supplementary material

Supplementary tables and figures
