# Supplementary material for: Inactivation of TRPM7 kinase in mice results in enlarged spleens, reduced T-cell proliferation and diminished store-operated calcium entry
Source: Sci Rep. 2018 Feb 14;8:3023. doi: 10.1038/s41598-018-21004-w (PMC5813043; doi:10.1038/s41598-018-21004-w)
Supplement: Supplementary file 1 — Supplementary tables and figures [file 41598_2018_21004_MOESM1_ESM.pdf]

Supplementary Data

Inactivation of TRPM7 kinase in mice results in enlarged spleens, reduced T-cell proliferation and diminished store-operated calcium entry

Pavani Beesetty[1], Krystyna B. Wieczerzak[1], Jennifer N. Gibson[1], Taku Kaitsuka[2], Charles Tuan Luu[1], Masayuki Matsushita[3] and J. Ashot Kozak[1]*

[1]Department of Neuroscience, Cell Biology and Physiology, Boonshoft School of Medicine, Wright State University, Dayton, OH 45435; [2]Department of Molecular Physiology, Faculty of Life Sciences, Kumamoto University, Kumamoto 860-8556, Japan; [3]Department of Molecular and Cellular Physiology, Graduate School of Medicine, University of the Ryukyus, Okinawa 903-0215, Japan.

*corresponding author
Biological Sciences Bldg. II, Rm. 251
Wright State University,
3640 Colonel Glenn Hwy.
Dayton, OH 45435

Supplementary Table S1: Complete blood counts with white blood cell differentials

Mouse ID	WT 1	WT 2	WT 3	KD 1	KD 2	KD 3
**White Blood Cells (K/µL)**	6.84	6.58	7.18	6.92	10.92	3.78
**Absolute Neutrophil Count (K/µL)**	0.92	0.8	1.01	0.63	1.53	0.66
**Absolute Lymphocyte Count (K/µL)**	5.24	5.18	5.75	5.79	8.45	2.87
**Absolute Monocyte Count (K/µL)**	0.4	0.45	0.34	0.48	0.69	0.15
**Absolute Eosinophil Count (K/µL)**	0.24	0.14	0.06	0.01	0.24	0.08
**Absolute Basophil Count (K/µL)**	0.04	0	0.02	0	0.01	0.01
**Neutrophil (%)**	13.4	12.18	14.11	9.16	14.05	17.57
**Lymphocyte (%)**	76.68	78.73	80.03	83.69	77.34	75.98
**Monocyte (%)**	5.81	6.85	4.78	7	6.29	4.09
**Eosinophil (%)**	3.56	2.17	0.85	0.12	2.2	2.03
**Basophil (%)**	0.56	0.07	0.24	0.04	0.12	0.33
**Hematocrit - HCT (%)**	42.1	41.8	40.3	42.6	41.7	36.8
**Red Blood Cells - RBC (M/µL)**	9.49	9.31	9.42	9.87	9.56	8.71
**Hemoglobin (g/dL)**	12.9	12.8	12.3	13.6	12.8	11.4
**Mean Corpuscular Volume - MCV (fL)**	44.4	44.9	42.8	43.2	43.6	42.2
**Mean Corpuscular Hemoglobin - MCH (pg)**	13.6	13.7	13.1	13.8	13.4	13.1
**Mean Corpuscular Hemoglobin Concentration - MCHC (g/dL)**	30.6	30.6	30.5	31.9	30.7	31
**RBC Distribution Width - RDW (%)**	12.5	13.3	13	14.1	14.1	19.3
**Red Cell Standard Deviation - RSD (fL)**	5.6	6	5.6	6.1	6.1	8.1
**Absolute Reticulocyte Count (K/µL)**	0	27	13.2	19.7	49.7	47.9
**Reticulocytes (%)**	0	0.29	0.14	0.2	0.52	0.55
**Absolute Platelet Count - PLT (K/µL)**	569	736	684	659	661	652
**Mean Platelet Volume - MPV (fL)**	6.3	6.4	6.5	6.4	6.3	6.5
**Platelet Distribution Width - PDW (%)**	41.4	43.1	41.1	43.1	41.9	43.4
**Plateletcrit - PCT (%)**	0.358	0.471	0.445	0.422	0.416	0.424
**Absolute Nucleated Red Blood Cell Count (K/µL)**	0	0	0	0	0	0
**Nucleated Red Blood Cell (%)**	0	0	0	0	0	0
**Result**	Platelet clumping, could be an artifact due to cardiac stick after euthanasia. No other significant findings.	No significant findings.	No significant findings.	No significant findings.	No significant findings.	No significant findings.

Supplementary Table S2:

A) Primer sequences

**Gene**	**Forward primer (5'-3')**	**Reverse primer (5'-3')**	**Product length**
TRPM7	CTGAAGCTGGGAAAATCAGC	ACCAAGTTCCAGGACCACAG	495
TRPM6	CCAGGTGCCGGTAATAACA	CTCTTGTGGCTGCCTTAGGT	220
TRPV1	CGAGGATGGGAAGAATAACTC	GGATGATGAAGACAGCCTTGA	188
STIM1	CCAGAGTCTCAGCCATAGTC	CTTCAGTACAGTCCCTGTCA	481
GAPDH (1)	GTGAAGGTCGGAGTCAACGGATTT	CACAGTCTTCTGGGTGGCAGTGAT	555
GAPDH (2)	AGGCCGGTGCTGAGTATGTC	TGCCTGCTTCACCACCTTCT	530

GAPDH (2) primers are used only in Fig. 4B.

B) EC_{50} (for Ca^{2+} and Mg^{2+})

**Condition**	**EC_{50} (mM)**	**EC_{50} - SE**	**Hill coefficient**	**Hill coefficient - SE**	**Adj R-square**
WT 24 hours – Ca^{2+}	0.175	0.005	3.04	0.383	0.983
KD 24 hours – Ca^{2+}	0.159	0.003	3.03	0.190	0.994
WT 24 hours – Mg^{2+}	0.065	0.010	2.62	0.814	0.959
KD 24 hours – Mg^{2+}	0.063	0.004	2.53	0.377	0.959
WT 48 hours – Ca^{2+}	0.130	0.002	6.96	0.317	0.947
KD 48 hours – Ca^{2+}	0.152	0.002	6.50	0.202	0.980
WT 48 hours – Mg^{2+}	0.039	0.002	1.08	0.058	0.983
KD 48 hours – Mg^{2+}	0.052	0.002	1.46	0.063	0.972

C) IC_{50} (For CsA and FK506)

**Condition**	**IC_{50} (nM)**	**IC_{50} - SE**	**Hill coefficient**	**Hill coefficient - SE**	**Adj R-square**
WT 48 hours - CsA	36.2	1.39	4.09	0.324	0.999
KD 48 hours - CsA	31.6	1.95	4.06	0.701	0.998
WT 48 hours - FK506	0.293	0.024	3.60	1.282	0.997
KD 48 hours - FK506	0.265	0.038	3.57	1.455	0.994

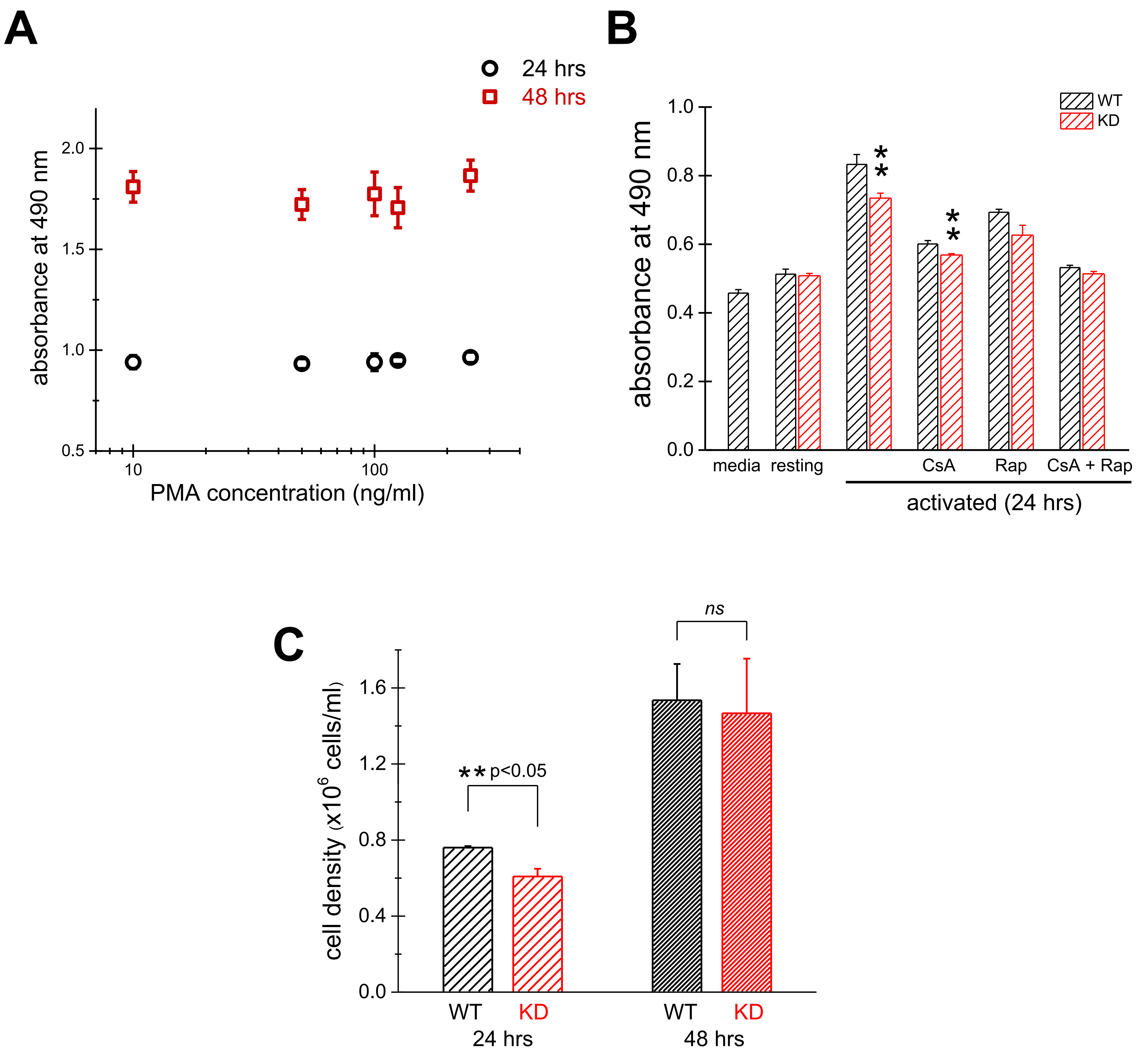

Supplementary Figure S1. ***Dependence of T-cell proliferation on PMA concentrations ranging from 10 to 250 ng/ml and immunosuppressant drugs CsA and rapamycin.*** A. Proliferation of WT T cells at indicated PMA concentrations (10, 50, 100, 125, 250 ng/ml) at a fixed ionomycin concentration of 250 nM was measured 24 and 48 hrs after activation using MTS-based proliferation assay. Proliferation at 48 hrs was higher than at 24 hrs but no significant differences were observed between concentrations of PMA tested. Data were collected from T cells isolated from two WT mice. B. 24 hrs after activation with 125 ng/ml PMA and 250 nM ionomycin, KD T cells showed more sensitivity to CsA inhibition compared to WT. The concentrations of CsA and rapamycin were 500 nM and 300 nM, respectively. C. Viable cell densities of WT and KD T cells 24 and 48 hrs after activation as in B in 96 well plate, measured using Neubauer haemocytometer. Data from 3 WT and 3 KD mutant mice. ** denotes $p<0.05$.

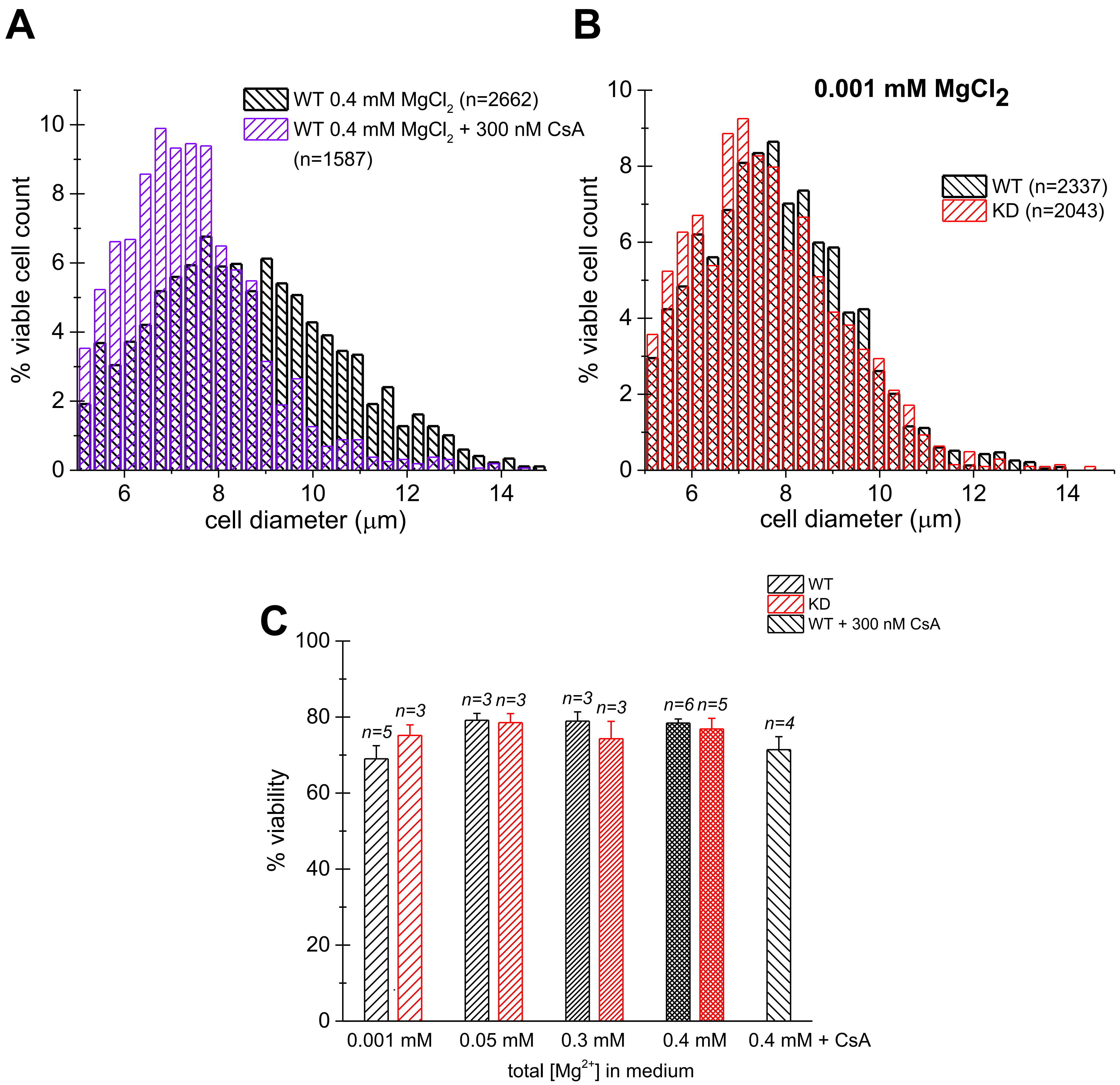

Supplementary Figure S2. ***Dependence of blastogenesis and proliferation on [Mg^{2+}]$_o$.*** A. Histogram showing the inhibition of WT T-cell blastogenic response by CsA. B. The blastogenic response is smaller in KD T cells than WT after 24 hrs of activation in the presence of 1 μM $MgCl_2$. C. Percent viability of WT and KD T cells activated under various concentrations of Mg^{2+} in the medium: no significant differences were observed at these Mg^{2+} concentrations or in the presence of CsA, tested by ANOVA. For A-C, WT and KD T cells were activated for 24 hrrs in Chelex-treated RPMI supplemented with 0.424 mM Ca^{2+} and the indicated Mg^{2+} concentrations. In panels A and B "n" indicates the number of cells. In panel C, "n" is the number of mice.

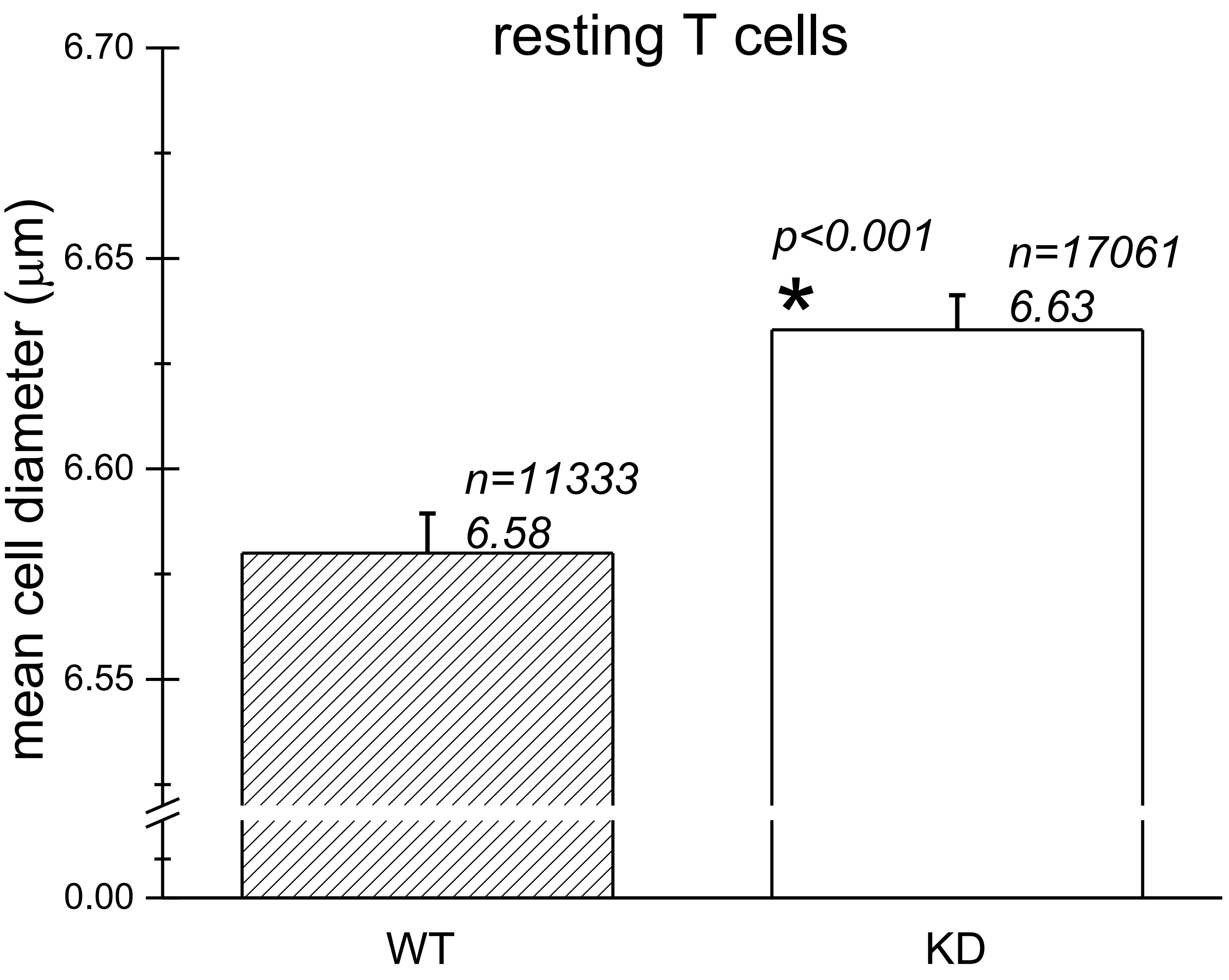

Supplementary Figure S3. ***Diameters of resting T cells in KD mice are larger than in WT.*** Diameters of resting T cells from WT and KD mouse spleens measured on the day of isolation. The mean diameter and volume of KD T cells were 0.76% and 2.5% larger than WT T cells, respectively. Data was collected from 5 WT and 5 KD mice in the age range of 13-18 weeks.

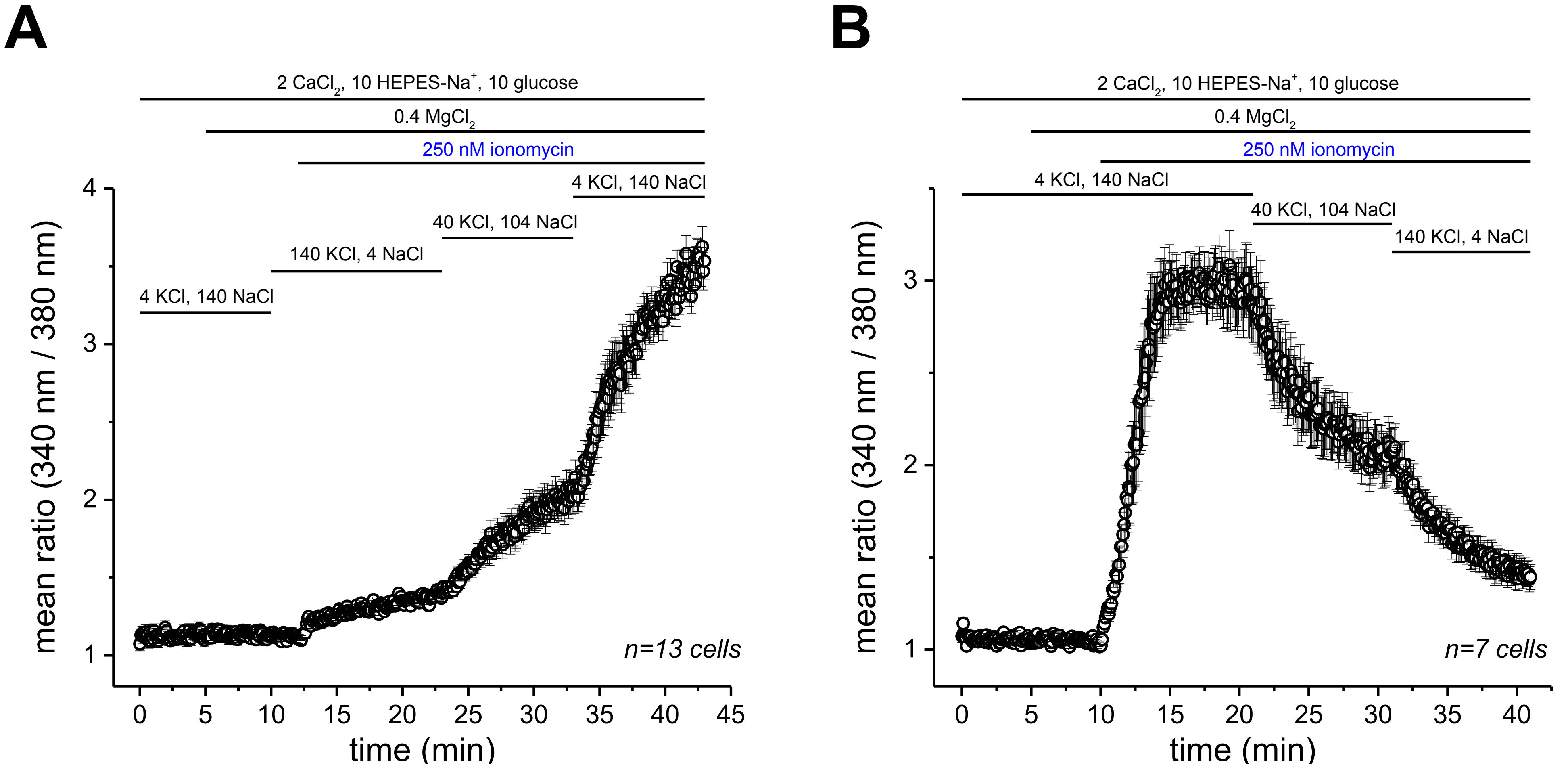

Supplementary Figure S4. ***Ionomycin-mediated intracellular Ca^{2+} elevation depends on the membrane potential.*** Fura-2 calcium imaging in WT splenocytes in the presence of 250 nM ionomycin (same concentration as in Suppl. Fig. 1) and varying the concentration of KCl . A. Hyperpolarizing the membrane potential by progressively decreasing $[KCl]_o$, increased $[Ca^{2+}]_i$ signal. B. Ca^{2+} levels decreased by depolarizing the membrane potential (by increasing $[KCl]_o$). The concentrations are in mM. $[Cl^-]$ was kept constant throughout the experiment.

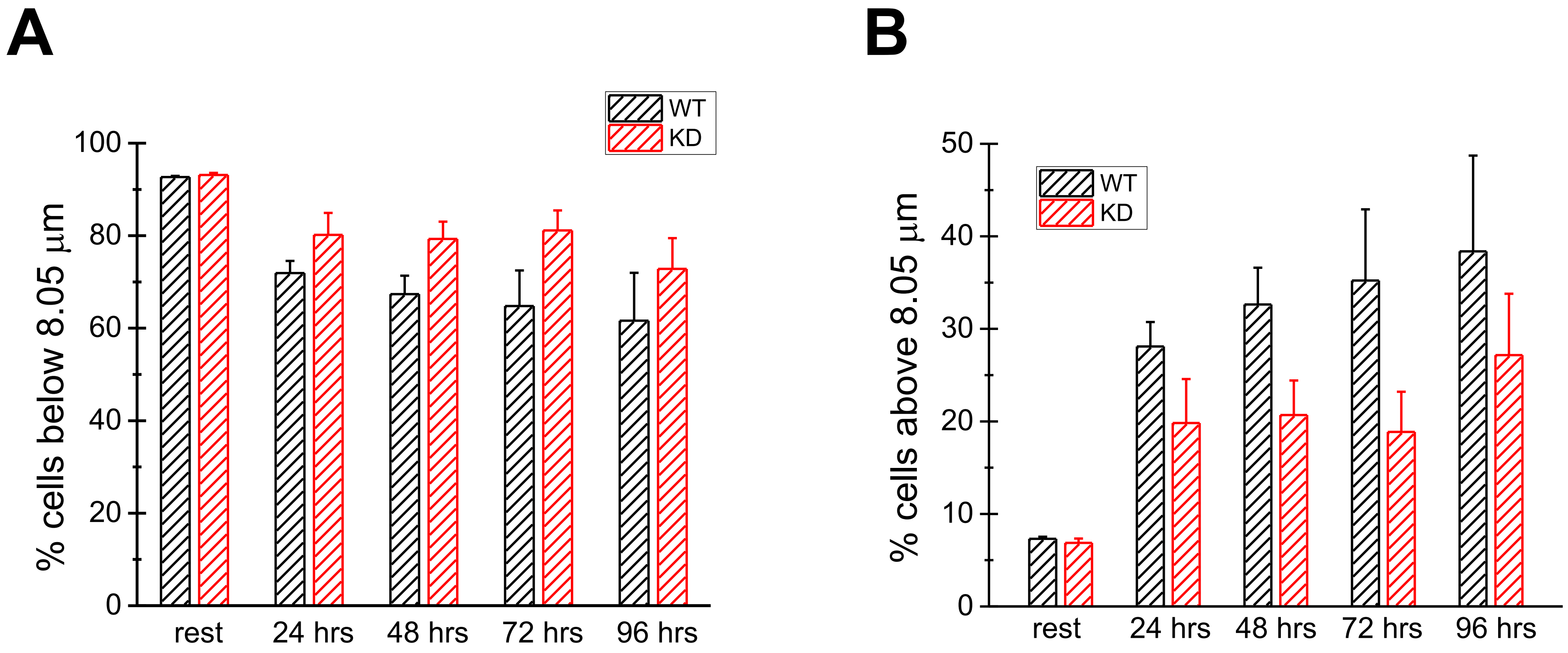

Supplementary Figure S5. ***Percentage of cells undergoing blastogenesis in response to anti-CD3/CD28 stimulation is lower in KD T cells.*** A. After activation with anti-CD3/CD28 coated beads, the percentage of cells with diameters below 8.05 µm was higher in KD compared to WT. B. The percentage of cells with diameters above 8.05 µm was lower in KD after activation. Data collected from the same 3 WT and 3 KD mice as in Figure 8.

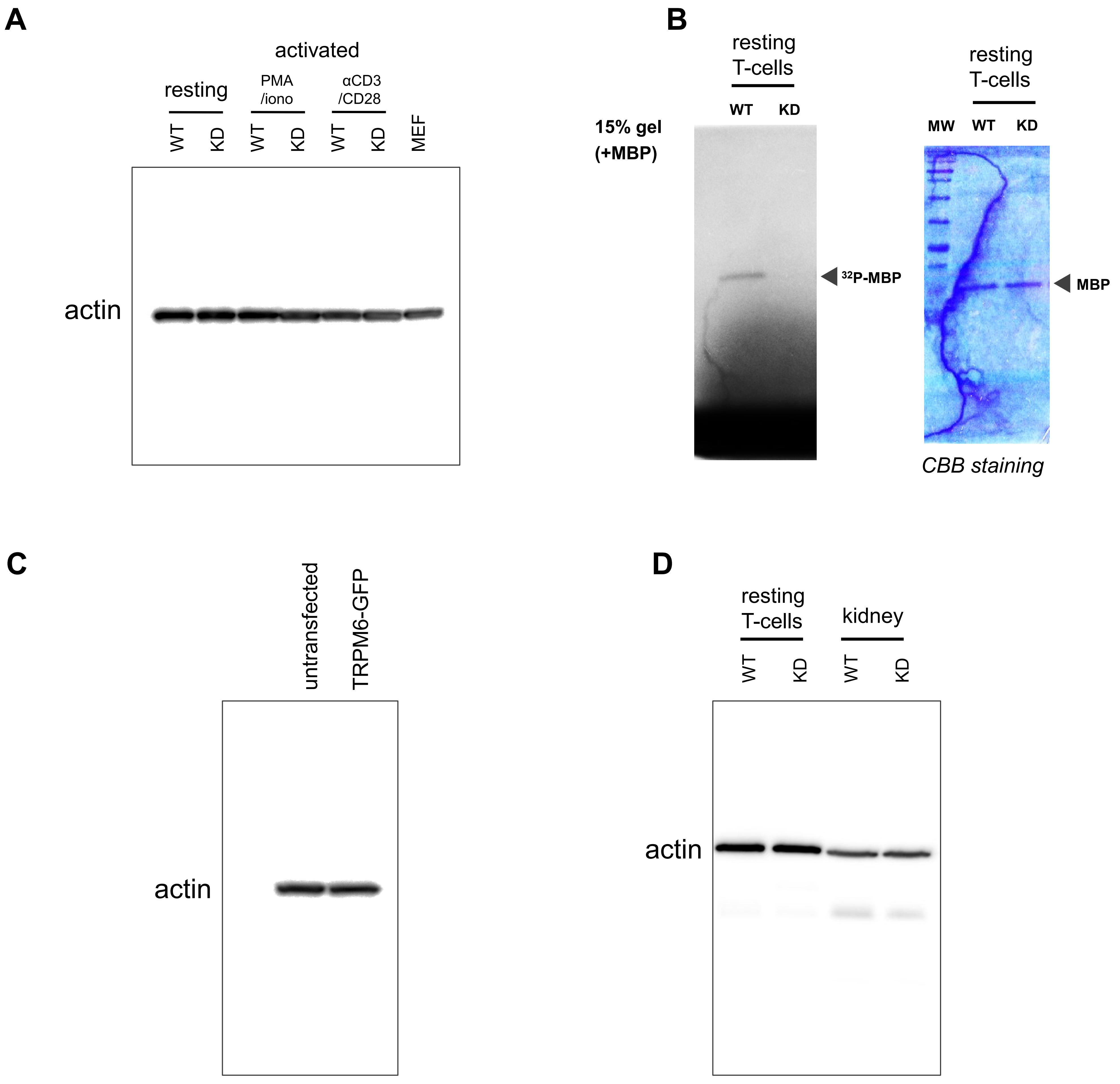

Supplementary Figure S6. ***Full-length gel images of Figure 5.*** A, C and D show the full-length gel images of actin bands in Fig 5 A, C and D, respectively. Whole cell lysate samples were run in A, C and D. The full-length gel images of Fig 5B are shown in B.
